# The Influence of Nucleus Mechanics in Modelling Adhesion-independent Cell Migration in Structured and Confined Environments

**DOI:** 10.1007/s11538-023-01187-8

**Published:** 2023-08-25

**Authors:** Chiara Giverso, Gaspard Jankowiak, Luigi Preziosi, Christian Schmeiser

**Affiliations:** 1 Department of Mathematical Sciences, Politecnico di Torino, Corso Duca degli Abruzzi 24, 10129 Torino, Italy; 2 Department of Mathematics and Statistics, University of Konstanz, 78457 Constance, Germany; 3 Faculty of Mathematics, University of Vienna, Oskar-Morgenstern Platz 1, 1090 Wien, Austria

**Keywords:** Cell migration, Mathematical modelling, Friction-based migration, Focal adhesion, Cytoskeleton

## Abstract

Recent biological experiments (Lämmermann et al. in Nature 453(7191):51–55, 2008; Reversat et al. in Nature 7813:582–585, 2020; Balzer et al. in ASEB J Off Publ Fed Am Soc Exp Biol 26(10):4045–4056, 2012) have shown that certain types of cells are able to move in structured and confined environments even without the activation of focal adhesion. Focusing on this particular phenomenon and based on previous works (Jankowiak et al. in Math Models Methods Appl Sci 30(03):513–537, 2020), we derive a novel two-dimensional mechanical model, which relies on the following physical ingredients: the asymmetrical renewal of the actin cortex supporting the membrane, resulting in a backward flow of material; the mechanical description of the nuclear membrane and the inner nuclear material; the microtubule network guiding nucleus location; the contact interactions between the cell and the external environment. The resulting fourth order system of partial differential equations is then solved numerically to conduct a study of the qualitative effects of the model parameters, mainly those governing the mechanical properties of the nucleus and the geometry of the confining structure. Coherently with biological observations, we find that cells characterized by a stiff nucleus are unable to migrate in channels that can be crossed by cells with a softer nucleus. Regarding the geometry, cell velocity and ability to migrate are influenced by the width of the channel and the wavelength of the external structure. Even though still preliminary, these results may be potentially useful in determining the physical limit of cell migration in confined environments and in designing scaffolds for tissue engineering.

**Supplementary Information:**

The online version contains supplementary material available at 10.1007/s11538-023-01187-8.

## Introduction

Cell migration on two dimensional (2D) substrates and inside three dimensional (3D) environments plays an essential role in many physiological and pathological processes, including embryonic development, wound healing, immune response, cancer progression and metastasis formation (Wolf and Friedl 2006; Bergert et al. 2015; Trepat et al. 2012). The unconfined motion of cells on 2D extracellular matrix (ECM) is a well-studied process and it is conventionally described by continuous and highly coordinated cyclic processes: the elongation of protrusions at the leading edge driven by actin polymerization, the formation of integrin-mediated focal adhesions (FAs), myosin-mediated contraction and the detachment of the trailing edge (Trepat et al. 2012; Balzer et al. 2012; Abercrombie et al. 1970). This classical description requires that specific transmembrane adhesion proteins (integrins, among others) carry intracellular forces from the cytoskeleton to the substrate to propel the cell forward (Bergert et al. 2015; Rafelski and Theriot 2004; Vicente-Manzanares et al. 2009) and it is therefore called *adhesion-dependent migration* or *integrin-mediated migration*.

While this mechanism of motion is well understood, the physical challenges that cells have to face when moving in 3D environments are only now receiving more attention, and recent researches indicate that in vivo cell migration can substantially deviate from migration on 2D unconfined substrates (Davidson et al. 2014; Balzer et al. 2012). Indeed, during motion through tissues, ECM barriers, capillaries and lymph nodes, cells experience varying degrees of physical confinement and cell migration can thus be achieved with very different mechanisms (Lämmermann et al. 2008; Balzer et al. 2012; Bergert et al. 2015; Even-Ram and Yamada 2005). In particular, it has been observed that cell migration in 3D environments can occur even in the absence of focal adhesions, suggesting that additional mechanisms for adhesion and migration are possible (Lämmermann et al. 2008; Reversat et al. 2020).

Such *adhesion-independent migration* has been observed in 3D confined environments (Lämmermann et al. 2008; Friedl and Bröcker 2000; Friedl et al. 2001; Fraley et al. 2010; O’Neill et al. 2018), using different cell lines and technologies. For cells of different types (dendritic cells in Lämmermann et al. (2008); leukocytes in Reversat et al. (2020); breast carcinoma, pancreatic carcinoma, and human osteosarcoma cells in Balzer et al. (2012)), it has been observed that migration in 3D environments may not require myosin-mediated contraction and that inhibitors of integrins do not hamper migration through channels leading to cell confinement, although these treatments can hinder and even prevent motility in wider channels leading to unconfined migration. Considering leukocyte migration (Reversat et al. 2020), on the one hand, it was shown that leukocytes do not migrate when confined between two parallel flat plates in the absence of adhesion. On the other hand, leukocyte adhesion-free motion is possible when supporting pillars or microfabricated structured channels are placed between these plates, under the conditions that the pillar size and spacing—or the characteristic length of the sidewall structure—match roughly that of the cell length (Reversat et al. 2020). No cell migration is observed when the cell is confined between flat parallel plates in two directions, using the experimental set-up and the cell line reported in Reversat et al. (2020).

Even though the origin and transmission of propelling forces during focal adhesion-free migration are not fully understood, all these findings (Lämmermann et al. 2008; Balzer et al. 2012; Reversat et al. 2020) indicate that 2D is essentially integrin-dependent and that adhesion-free motility relies on a structured physical confinement, only achievable in a 3D setting, that can induce cytoskeletal alterations reducing the dependence of cell motion on the adhesion-contraction force coupling. In the absence of adhesions, non-specific transient interactions between transmembrane proteins and the substrate could generate friction that converts protrusive actin cortex flow into cell movement (Bergert et al. 2015; Hawkins et al. 2011; Lämmermann et al. 2008). It has also been observed that confined migration depends largely on microtubule (MT) dynamics and might persist even when F-actin is disrupted (Balzer et al. 2012; Stroka et al. 2014; Li and Sun 2018).

Although increasing levels of confinement can trigger transitions from integrin-based towards adhesion-independent migration modes in many cell types (Friedl et al. 2001), in the absence of matrix protease production, a too strong confinement either decreases or even prevents migration, due to cell stiffness (Wolf et al. 2003, 2013). In particular, while the cytoplasm is very flexible and the cytoskeleton can actively remodel to undergo large deformations and penetrate small openings, the cell nucleus is normally 2–10 times stiffer than the surrounding cytoplasm and, with a typical diameter of 3–10 $\upmu$m, occupies a large fraction of the cellular volume and is usually larger than many of the pores encountered in the extracellular environment (Davidson et al. 2014; Wolf et al. 2013; Cao et al. 2016). Thus, the nucleus should undergo substantial deformations when the cell moves through 3D constrictions, and it may constitute a rate-limiting factor during non-proteolytic migration of cells (Davidson et al. 2014; Wolf et al. 2013).

To understand the bio-physical and mechanical factors involved in the process of cell migration, many mathematical models have been proposed in the past decades (Jilkine and Edelstein-Keshet 2011; Holmes and Edelstein-Keshet 2012; Dreher et al. 2014; Danuser et al. 2013). Specifically, there have been abundant works related to cell migration on 2D substrates, either modelling the membrane mechanics and its signalling activity (Elliott et al. 2012; Hecht et al. 2011) or describing in detail the cytosol dynamics (Shao et al. 2010; Recho et al. 2013, 2015; Manhart et al. 2015). However, coupled models, including the cytosolic machinery and membrane dynamics, have received little attention, even though they are critical to understand cell migration (Dreher et al. 2014; Danuser et al. 2013; Giverso and Preziosi 2018; Moure and Gomez 2018). Furthermore, most of these models have focused on 2D adhesion-dependent cell motility, in which cells extend a stationary lamellipodium at the leading edge. Even in models accounting for amoeboid motion and which have been extended to model 3D confined migration (Moure and Gomez 2016, 2017, 2018), the cell motility substantially relies on adhesion, on acto-myosin protrusion-contraction, and on cell capability to sense an external field through membrane receptors. On the contrary, adhesion-independent migration inside constrained 3D environments has received less attention and mathematical models have started to tackle this interesting mechanism only recently. In particular, a simplified two-dimensional model for focal adhesion-independent cell swimming, based on the flow-friction driven force transmission, has been proposed by Wu et al. (2018) and Stotsky and Othmer (2022), while in Kaoui et al. (2008) the motion of closed phospholipid membranes suspended in a nonlinear shear gradient of a plane Poiseuille flow was investigated numerically in two dimensions. A possible explanation of the chemical signalling activity regulating adhesion-independent migration has been advanced in Elliott et al. (2012), using a system of reaction-diffusion equations and assuming a Turing instability to model a polymerization pattern on the cell surface, which drives the formation of pseudopods. We remark that the models for cell motion that introduce a friction coefficient between the cytoskeleton flow and the substrate to represent adhesion (Tawada and Sekimoto 1991; Giverso and Preziosi 2018; Farutin et al. 2019; Chelly and Recho 2022; Loisy et al. 2019) could as well be used to describe non-specific sliding friction (Farutin et al. 2019). Indeed, even though conceived for modelling the specific integrin-based adhesion, they can be adapted to describe transient interactions between the membrane and the substrate or the surrounding fluid, by performing appropriate calibration of the friction term. Recently, a couple of mathematical models have also investigated the non trivial limit of a vanishing friction coefficient with respect to other internal dissipative processes (Chelly and Recho 2022; Loisy et al. 2019; Le Goff et al. 2020), demonstrating that motility can still occur and making the models non-specific to the adhesion properties of the cell with its environment. However, since most of these models are interested in determining the minimal ingredient for the onset of cell motion, they are solved in a 1D setting (Farutin et al. 2019; Loisy et al. 2019; Le Goff et al. 2020). Furthermore, in all these cases (Kaoui et al. 2008; Wu et al. 2018; Stotsky and Othmer 2022; Elliott et al. 2012; Moure and Gomez 2017, 2018; Chelly and Recho 2022; Loisy et al. 2019; Le Goff et al. 2020), the presence of the nucleus as a limiting factor for cell migration and the effect of confinement are not taken into account.

The influence of nuclear deformations on the whole process of cell migration was included in some recent works. In Moure and Gomez (2020) the role of the cell nucleus was studied using a computational model of a fish keratocyte, but the model is specifically conceived for 2D cell migration and thus it cannot be used for 3D confined migration. On the other hand, Cao et al. (2016) develop a chemo-mechanical model to study the nuclear strains and shapes, its plastic deformation and the threshold for the rupture of its envelope during migration through confined interstitial spaces. In Lee et al. (2017), a 2D model for cell migration through a dense network of host cells was proposed to reproduce glioma cell invasion. The moving cell is represented by two elastic closed curves, an inner curve corresponding to the nucleus of the cell and an outer curve corresponding to the cell basal membrane, whereas non-moving cells are represented by a single elastic curve. In Chen et al. (2018), the deformations of the cell and nucleus during invasion through a dense microenvironment were simulated incorporating stochastic processes and uncertainties in the input variables were evaluated using Monte Carlo uncertainty quantification simulations. These models Lee et al. (2017), Chen et al. (2018) and Cao et al. (2016) were able to reproduce correctly the hourglass cell and nucleus deformation observed in biological experiments, by relying on an external chemical factor.

In this work, we build on top of Jankowiak et al. (2020) to develop a simplified framework to study whether adhesion-free migration could be driven by simple mechanical features. We also test the influence of cell nucleus mechanical properties in the determination of the physical limit of cell migration. Thus, we propose a model of force generation during adhesion-independent cell migration in confined environments, taking into account the flow-friction driven force transmission, the cell membrane polymerization and the nuclear deformations. The cell is modelled by two membranes, an outer one representing the cell membrane and an inner one representing the nucleus. The two membranes are connected by microtubules, responsible for the nucleus location inside the cell. The renewal of the actin network underneath the cell membrane is modelled by the evolution of the mass distribution along the membrane, with a source (resp. sink) term at the front (resp. back), while also taking into account the conservation of the centre of mass. The model is used to simulate cell motion inside channels with structured walls with wavelengths ranging in the order of magnitude of the cell and nucleus diameters. We present the mathematical model in Sect. 2 and the numerical scheme we used to solve the systems of equations in Sect. 3. Finally, in Sect. 4, we present and discuss the numerical results.

## The Mathematical Model

In this section, we present the continuum model ingredients for adhesion-free cell migration in domains containing rigid obstacles with a given geometry. Motivated by the experimental setup of Reversat et al. (2020), where the cell is confined between flat top and bottom surfaces and structured side walls, we choose a two-dimensional model, representing the projection of the three-dimensional set-up along the vertical directions, i.e. the one perpendicular to the flat walls. We consider the cell composed by two main compartments, the cytoplasm and the nucleus, both surrounded by membranes. The nuclear and cellular membranes can be represented as closed curves of ${\mathbb {R}}^2$. We identify the cell cortex with the cell membrane, and describe their ensemble as a single curve. This means that we do not model detachment and reattachment events between the cortex and the membrane, which are known to occur in certain cells. The cell cortex is schematically represented by a lipid bilayer and a complex underlying network of actin filaments. It is assumed to be elastic and subjected to a pressure differential force acting in its outward normal direction. The renewal of the actin network composing the cellular cortex is a key ingredient during many cellular behaviours and is here modelled by deposit and removal of material along the cortex. In rough biological terms, this corresponds to an imbalance between polymerization and depolymerization of some parts of the actin filaments and gives the cell a preferential direction of movement. The actin is then transported in the cell to a new location where it polymerizes again (actin treadmilling). This transport mechanism needs to be taken into account to ensure conservation of momentum in the absence of external forces. In our model, this is done by including an additional reaction force, as detailed in Sect. 2.2. All these mechanical contributions on the cortex balance with frictional effects from the surrounding fluid (both external and internal to the cell), which are not explicitly described.

Finally, the nuclear membrane can be thought of as a double phospholipid bilayer with an associated mesh of intermediate filaments forming the nuclear lamina which stabilises the nucleus and provides some resistance to bending and tension. The nuclear envelope is eventually subjected to the differential pressure between the cytosol and the interior of the nucleus. Also in this case, we represent the ensemble of the nuclear membrane and lamina with a single curve. We use either the term nuclear envelope or nuclear membrane to refer to the whole structure of membranes and lamins surrounding the nucleus, as well as either the term cell membrane or cell cortex denote the outer membrane with associated actin cortex.

### Description of the Model and Notation

Since we consider 2D-projections along the directions perpendicular to the flat plates of the 3D set-up used in Reversat et al. (2020), we set ourselves in ${\mathbb {R}}^2$ and consider the cell cortex and the nuclear membrane represented by the time-dependent closed curves $\Gamma (t)$ and $\Gamma _n(t)$ respectively,

$$\begin{aligned} \Gamma (t) = \left\{ X(t,s) : s\in {\mathbb {T}}_M\right\} , \text { where } X(t, s) : [0, T] \times {\mathbb {T}}_M \rightarrow {\mathbb {R}}^2\,, \\ \Gamma _n(t) = \left\{ Y(t,\sigma ) : \sigma \in {\mathbb {T}}_1\right\} , \text { where } Y(t, \sigma ) : [0, T] \times {\mathbb {T}}_1 \rightarrow {\mathbb {R}}^2\,, \end{aligned}$$

where $T > 0$ is some fixed maximal time and $M > 0$ is the fixed total amount, or mass, of actin along the membrane. The space variable *s* (resp. $\sigma$) belongs to ${\mathbb {T}}_M = {\mathbb {R}} / M{\mathbb {Z}}$, which can be thought of as the interval [0, *M*] where 0 and *M* are identified, so that, for any fixed $t\in [0,T]$, the continuity of $X(t,\cdot )$ and $Y(t,\cdot )$ is enough to enforce the closedness of the curves $\Gamma (t)$ and $\Gamma _n(t)$. Note that *X* and *Y* are not assumed to be arc length parameterizations. The time derivatives are denoted by $\partial _t X$ and $\partial _t Y$ whereas the space derivatives (along the curves) are $\partial _s X$ and $\partial _{\sigma } Y$, respectively. In some sense, the variable *s* counts the mass of actin along the curve and therefore tracks Lagrangian particles. More precisely, for any non empty interval $[s_1, s_2]$ with $0 \le s_1 < s_2 \le M$, the amount of actin on the corresponding piece of cortex, i.e. between $X(t, s_1)$ and $X(t, s_2)$, is $\vert s_2 - s_1\vert$. Therefore, *X* encodes not only geometric information (the shape of the curve), but also information about the distribution of actin inside the cortex. By writing the problem in terms of *s* (as opposed to arc length), we can describe the time evolution of both these quantities with a single equation for *X*. Writing the equation in terms of the arc length $\ell \in [0, L]$ makes it harder to deal with a time-dependent length *L*, as is the case here, especially for the numerics. Therefore, we work with the time independent ${\mathbb {T}}_M \ni s$. For the sake of completeness, let us highlight the link between the variable *s*, the arc length $\ell$ and the actin density on the cortex, which we will denote by $\rho$. As is standard, the length between two points $X(t, s_1)$ and $X(t, s_2)$ is given by

$$\begin{aligned} L(t, s_1, s_2) = \int _{s_1}^{s_2} \vert \partial _s X(t, s)\vert \; ds. \end{aligned}$$

As already mentioned, the mass of actin between $X(t, s_1)$ and $X(t, s_2)$ is $\vert s_2~-~s_1\vert$, so that, if one assumes that $s \mapsto \vert \partial _s X(t, s)\vert \in L^1({\mathbb {T}}_M)$ is positive, we have by Lebesgue differentiation theorem that

$$\begin{aligned} \rho (t, s_1) = \lim _{s_2 \searrow s_1} \frac{\vert s_2 - s_1 \vert }{L(t, s_1, s_2)} = \lim _{s_2 \searrow s_1}\frac{\vert s_2 - s_1 \vert }{\int _{s_1}^{s_2} \vert \partial _s X(t, s)\vert \; ds} = \vert \partial _{s_1} X(t, s_1) \vert ^{-1}. \end{aligned}$$

If we now consider *s* to be a function of the arc length $\ell$, we have, with a slight abuse of notation, that

1 $$\begin{aligned} s(t, \ell ) = \int _0^\ell \rho (t, l)\;dl , \end{aligned}$$

so that $\frac{\partial s}{\partial \ell } = \rho (t, \ell ) = \big \vert \partial _s X(t, s(t, \ell ))\big \vert ^{-1}$. By a change of variables, this is compatible with

2 $$\begin{aligned} \ell (t, s) = L(t, 0, s) = \int _0^s \vert \partial _s X(t, {\tilde{s}})\vert d{\tilde{s}} . \end{aligned}$$

In the following, we take $M=1$, implying that the actin mass is normalised with respect to a reference total mass, so that $s \in {\mathbb {T}}_1$.

**Fig. 1:**
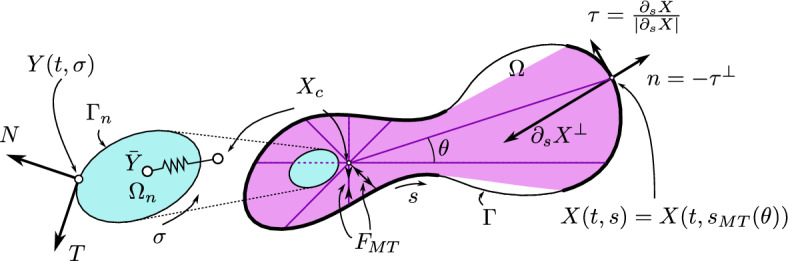
The parameterization and associated vector quantities, along with the representation of the microtubule network geometry, with the centrosome $X_c$ linked to the centroid of the nucleus ${\bar{Y}}$. The pink region shows where microtubules are present, the corresponding anchoring points on the membrane are represented in bold stroke. The bottom arrows indicate the orientation of the curves. The elastic link between the centrosome and the nucleus (coloured in cyan) is also represented, as well as the microtubular force $F_{MT}$, both of which will be detailed later on (Color figure online)

Assuming that the curves are smooth enough, we denote by $\tau (t, s)$ and *n*(*t*, *s*) the unit tangent and unit outward normal vectors to the cell membrane curve at *X*(*t*, *s*) and with $T(t, \sigma )$ and $N(t, \sigma )$ the unit tangent and unit outward normal vectors to the nuclear membrane curve at $Y(t, \sigma )$. Assuming positive orientation of the parametrization, we have

3 $$\begin{aligned} \tau = \frac{\partial _s X}{\vert \partial _s X\vert } ,\qquad n = -\tau ^\bot , \qquad T = \frac{\partial _\sigma Y}{\vert \partial _\sigma Y\vert } ,\qquad N = -T^\bot , \end{aligned}$$

with the convention $(a,b)^\bot = (-b,a)$. A sketch of the notations employed in the paper is illustrated in Fig. 1.

Finally, let $\Omega (t) \subset {\mathbb {R}}^2$ be the set bounded by $\Gamma (t)$ and $\Omega _n(t) \subset {\mathbb {R}}^2$ the set bounded by $\Gamma _n(t)$, i.e. $\Gamma (t) = \partial \Omega (t)$ and $\Gamma _n(t) = \partial \Omega _n(t)$: for biological consistency we have to guarantee that the nucleus is located inside the cell, i.e. $\Omega _n \subset \Omega$. During cell motion and other biological processes (e.g. development, mitosis, fertilisation, ...), the positioning of the nucleus is of paramount importance in the establishment of cellular architecture (Tran et al. 2001). Nuclear positioning is generally dependent on some cytoskeleton constituents, mainly microtubules (MTs), which are dynamic polymers of tubulin, and intermediate filaments, composed of a family of related proteins having common sequence and structural features. Microtubules originate from the MT-organising centre (MTOC), with functions that include microtubule nucleation, stabilisation, and/or anchoring. The best-studied MTOC is the centrosome, which is found in many animal cells. The centrosome is connected to the cell nucleus through MTs, and associated intermediate filaments forming a ring network around the nuclear envelope, as well as to the cell actin cortex, via MTs. Thus, the MT structure and its related centrosome couple the nucleus to the cellular envelope, and play a fundamental role in providing structure and shape to cells, in determining cell migration direction and persistence, and in locating the cell nucleus (Fruleux and Hawkins 2016; Gundersen and Worman 2013; Beadle et al. 2008; Friedl et al. 2011). Microtubules constantly switch between growing and shrinking states, through assembly and disassembly of tubulin monomers at their ends, in a process termed dynamic instability. They are able to generate pushing forces over the cell membrane during their assembly process, and pulling forces during their disassembly process (Laan et al. 2008). In this work, we disregarded the description of the dynamic growth/shrinkage of the MTs through the addition and detachment of monomers, but the MT structure is built at each time *t*, depending on the position of the centrosome and the cell cortex. In this scenario, without describing the incremental growth of each filament over time, the MT structure evolves and it is not fixed once and for all. By adopting a continuous setting, at each instant of time, the MTs are assumed to be homogeneously distributed around the centrosome, located in $X_c(t)$, to all points on the cortex that can be connected to the centrosome by a line segment, lying inside the cell. The region of the cell in which MTs can be defined is coloured in pink in Fig. 1.

With that in mind, it is possible to define, for every time *t*, the microtubule anchoring points on the membrane cortex as the first intersection of the half line starting at $X_c(t)$ with angle $\theta \in [0, 2\pi )$ with the cell cortex. Formally, we define the map

$$\begin{aligned} \Pi _{MT}(t, \theta ) = X_c(t) + \lambda _{MT}(t, \theta ) \textrm{e}_{\theta } , \end{aligned}$$

where

$$\begin{aligned} \lambda _{MT}(t, \theta ) := \min \left\{ \lambda \ge 0: X_c(t) + \lambda \textrm{e}_{\theta } \in \Gamma \right\} \text { and } \textrm{e}_{\theta } = \begin{pmatrix} \cos (\theta ) \\ \sin (\theta )\end{pmatrix}. \end{aligned}$$

This map $\Pi _{MT}$ is well defined if $X_c(t) \in \Omega (t)$ and is surjective if $\Omega (t)$ is star-shaped with respect to $X_c(t)$. Then, the cortex anchoring point of the microtubule located at an angle $\theta$ is defined as

4 $$\begin{aligned} X(t, s_{MT}(t, \theta )) {:}{=} \Pi _{MT}(t, \theta ). \end{aligned}$$

In Fig. 1, the portion of the cortex on which MTs can anchor is highlighted in bold stroke. Note that, with a slight abuse of notation, $\Pi _{MT}$ can also be seen as a map onto ${\mathbb {T}}_1$ such that $\Pi _{MT}(t,\theta ) = s_{MT}(t,\theta )$.

Then, the segment $[X_c(t), X(t, s_{MT}(\theta ))]$ represents a microtubule at a given instant of time. The construction of some representative MTs is illustrated by the purple segments in Fig. 1. We remark that MTs can be drawn in the whole region of the cell coloured in pink in Fig. 1 defining a continuous MT structure. Therefore the MTs structure homogeneously spans all the angles around the centrosome, and the distribution of the length of MTs can vary over time depending on the location of the centrosome and the cell membrane points. Since the 2D representation used in this paper stands for a projection of the 3D cell along the vertical direction, we assume that the MTs and the centrosome can be built also in the nucleus region (cyan area in Fig. 1), representing filaments extending either underneath or above the nucleus. Finally, we observe that in this description we disregarded the deformation of MTs and we cannot capture the MTs’ bending at the cell membrane (Geisterfer et al. 2020). However, the description of such phenomena will require a deeper mechanical characterisation of the MTs and will call for a 3D model in order to correctly represent the geometry of the MT filaments.

In the following, under the setting depicted above, we will derive the equations describing the evolution of the cell cortex (Sect. 2.2), the MTs structure (Sect. 2.3), and the nucleus membrane (Sect. 2.4). The dependence of the different dependent variable functions on the independent variables in their arguments will be omitted whenever possible.

### Evolution of the Cell Membrane

Concerning the evolution of the cell membrane, we refer to the model proposed in Jankowiak et al. (2020), properly modified in order to take into account the presence of the nucleus and the MT structure. The evolution of the actin density, describing the active component of the model due to the heterogeneity of the polymerization rate across the cortex, is given by Jankowiak et al. (2020):

5 $$\begin{aligned} \partial _t \rho (t, \ell ) = f(t, \ell ), \end{aligned}$$

where $f(t,\ell )$ is the rate of actin density increase ($f(t,\ell )>0$) or decrease ($f(t,\ell )<0$).

As done in Jankowiak et al. (2020), we assume that the total amount of actin in the cortex is kept constant and that the cell polarization and subsequent actin polymerization manifests itself by a local imbalance producing a net increase of actin density close to the cell front, balanced by a decrease close to the rear of the cell. Since it is assumed that the total amount of actin in the cortex does not change in time, we require

$$\begin{aligned} \int _{\Gamma } f(t,\ell ) \,d\ell = {{\int _{{\mathbb {T}}_1}}}f(t, \ell (t,s))\vert \partial _s X(t,s)\vert ds = 0 ,\qquad t\ge 0. \end{aligned}$$

The mass transfer rate, or polymerization rate, $r_\textrm{pol} \ge 0$ is then defined as

6 $$\begin{aligned} r_\textrm{pol} := \int _\Gamma f^+\; d\ell = -\int _\Gamma f^- \; d\ell , \end{aligned}$$

where $\left( \,\cdot \,\right) ^\pm$ denotes the positive and negative parts, respectively.

**Fig. 2:**
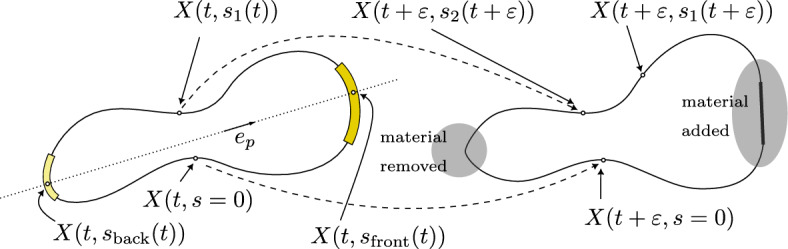
Left: Parameterization of the cell at time *t*. The front and back of the cell are shown, with the corresponding polymerization regions for a possible choice of *f*. The dark yellow and light yellow area highlight the support of ${f}^+$ and ${f}^-$, respectively. Right: Parameterization at time $t+\varepsilon$. The dashed lines show how the material points at *t* and $s=0$ and $s=s_1$ are mapped at $t+\varepsilon$. Because of the polymerization, $\partial s/\partial t > 0$ in the upper part of the curve between the two yellow regions, so that, at $t+\varepsilon$, the material point which was located at $X(t, s_1(t))$ is at $X(t+\varepsilon , s_2(t + \varepsilon ))$, for some $s_2 > s_1$ (Color figure online)

In practice, we assume that the cell is polarized in a given direction $e_p \in {\mathbb {S}}^1$, and that for each time *t*, there are unique $s_\textrm{back}(t)$, $s_\textrm{front}(t) \in {\mathbb {T}}_1$ (see the left of Fig. 2) so that

$$\begin{aligned} X(t, s_\textrm{back}(t)) \cdot e_p(t) = \min _{ s \in {\mathbb {T}}_1} X(t, s) \cdot e_p, \quad X(t, s_\textrm{front}(t)) \cdot e_p(t) = \max _{s \in {\mathbb {T}}_1} X(t, s) \cdot e_p. \end{aligned}$$

A reasonable choice for *f* is then a function with its (non negative) maximum at $X(t,s_\textrm{front}(t))$ and (non positive) minimum at $X(t,s_\textrm{back}(t))$.

For what concerns the MT endpoints density on the cell cortex, we can define

7 $$\begin{aligned} \begin{aligned} \rho _{MT}({t,}s)&= \left| (\Pi _\text {MT}^{-1})'({t,}s)\right| \\&= {\left\{ \begin{array}{ll} \left| \frac{d \theta }{d s} \right| = \left| \partial _s X \right| \dfrac{n\cdot (X - X_c)}{\vert X - X_c\vert ^2} &{} \text {if }X({t,}s) \in \Pi _{MT}({t,}[0, 2\pi )) \\ 0 &{} \text {otherwise.} \end{array}\right. } \end{aligned} \end{aligned}$$

To derive Eq. (7), we exploit the fact that $\dfrac{d\ell }{ds} = \vert \partial _s X\vert = \rho ^{-1}$ and that

$$\begin{aligned} \frac{dX}{d \theta } \cdot \textrm{e}_\theta ^\perp = \vert X - X_c\vert \Leftrightarrow \frac{dX}{d \theta } \cdot (X-X_c)^\perp = \vert X - X_c\vert ^2, \end{aligned}$$

leading to

$$\begin{aligned} \frac{d \theta }{d\ell } = \frac{n\cdot (X - X_c)}{\vert X - X_c\vert ^2}. \end{aligned}$$

Then, the evolution of the cell cortex is determined by the Newton’s Second Law, written in an overdamped regime, i.e. the friction of the surrounding fluid balances with all other contributions, leading to the following balance law for *X*:

8 $$\begin{aligned} \dfrac{D}{Dt} X- k_\tau v(\partial _t X_c, \omega , \theta ) \rho _{MT} = - F_{MT} \rho _{MT} - F_\textrm{cont} + F_\textrm{wall}+ F_{\textrm{comp}}+ F_{c} , \end{aligned}$$

where the friction coefficient in front of the time derivative is set equal to unity, without loss of generality, by an appropriate choice of the time scale, as done in Jankowiak et al. (2020). We remark that the description of the motion of the fluid both inside and outside the cell is here neglected. This assumption seems reasonable, at least referring to the biological experiments performed by Reversat et al. (2020), since we study the motion of a cell confined inside a channel where the hydrodynamic interactions are not the leading cause of cell motion. In Eq. (8), the velocity of the cell cortex relative to the laboratory coordinates is given by the material derivative *DX*(*t*, *s*)/*Dt* and not by the partial derivative $\partial X(t, s)/\partial t$. This is because of the implicit dependence of *s* on *t* which is introduced by the time evolution of the density $\rho$ in (5). In Fig. 2, we sketch why, for a nonzero polymerization rate $r_\textrm{pol}$ and for fixed *s*, *X*(*t*, *s*) does not track a material point. The material derivative is then defined as

9 $$\begin{aligned} \dfrac{D}{Dt} X(t,s)&= \partial _t X(t,s) + \frac{\partial s}{\partial t} \partial _s X(t,s)\, \nonumber \\&= \partial _t X(t,s) + \left( \int _0^{\ell (t, s)} f(t,\lambda )d\lambda \right) \partial _s X(t,s)\, \nonumber \\&= \partial _t X(t, s) + \left( \int _0^s f(t, \ell (t, {\tilde{s}})) \vert \partial _s X(t, {\tilde{s}})\vert d {\tilde{s}}\right) \partial _s X(t,s)\, \nonumber \\&= \partial _t X(t, s) + F_T(t,s)\,, \end{aligned}$$

where we used (1) and (5).

The second term on the l.h.s. and the first term in the r.h.s. of eq. (8) represent, respectively, the friction force generated by microtubule-binding complexes sliding on the cortex and the in-line force due to MTs elongation (as they will be detailed in Sect. 2.3). Both terms act on the portion of the cell membrane where MT anchor points can be defined and therefore they are weighted by $\rho _{MT}$, defined by Eq. (7). The second and third terms on the r.h.s. of Eq. (8) take into account, respectively, the contact between the cell cortex and the nucleus (see Sect. 2.4 for a detailed description of $F_\textrm{cont}$) and the one between the cell and the channel wall. In the following, we assume that $F_\textrm{wall}$ derives from a potential, a possible choice of which is proposed in Sect. 4. The compensating force $F_\textrm{comp}$ in the fourth term on the r.h.s. of Eq. (8) must be chosen (for each time *t*) so that the centre of mass is fixed if the effects of the confinement, nucleus and microtubule structure are removed and if the motion of actin along the cortex is perfectly balanced by the treadmilling, without any internal dissipation (see Jankowiak et al. (2020) for more details on the derivation). In this case, we obtain after integration

10 $$\begin{aligned} \begin{aligned} {{\int _{{\mathbb {T}}_1}}}F_{\textrm{comp}}(s) \; ds&= {{\int _{{\mathbb {T}}_1}}}F_T(s) \; ds \\&= -{{\int _{{\mathbb {T}}_1}}}X(s) f(\ell (s)) \vert \partial _s X(s)\vert \; {ds} = -\int _\Gamma X(s(\ell )) f(\ell ) \; d \ell , \end{aligned} \end{aligned}$$

where $F_T(s)$ is defined through Eq. (9) and it is related to the actin transport due to polymerization and depolymerization. Finally, the last term on the r.h.s. of Eq. (8) takes into account the forces acting on the cell due to the cortex mechanical behaviour and the pressure difference in and out of the cell and it can be obtained from the cell membrane energy

$$\begin{aligned}F_{c}=- \dfrac{\delta E_c}{ \delta X} \,, \qquad E_{c}= E_{\textrm{el}}+E_{\textrm{p}}\,.\end{aligned}$$

The membrane energy $E_c$ comprises an elastic term representing the cell and cell cortex elasticity $E_{\textrm{el}}$ and a term related to the existence of a differential pressure $E_{\textrm{p}}$. More precisely, the associated elastic energy $E_{\textrm{el}}$ is composed of two parts, the first one $E_{\textrm{el}}^{(1)}$ is related to the response of the cell membrane to tension and the second one $E_{\textrm{el}}^{(2)}$ is related to the response of the cell to deviations from its target area. The introduction of an elastic constraint on the cell membrane length and cell area is in agreement with prototypical models of membrane and cortex elasticity using elastic springs linking different parts of the cell (Barnhart et al. 2010; Du et al. 2012; Recho and Truskinovsky 2013; Kuchnir Fygenson et al. 1997). We observe that the elastic response of a cell may be associated with both the actin cortex and the phospholipid cell membrane. Since the actin cortex undergoes a constant renewal over a timescale of 30–100 s (Rubinstein et al. 2009) and bulk elastic stresses inside the cortex are relaxed over a time-scale of 1–10 s (Rubinstein et al. 2009; Mofrad 2009; Recho et al. 2015), which are much shorter than the characteristic timescale of motility experiments, the elastic behaviour of the outer curve $\Gamma$ is mainly associated with the mechanical response of the cell membrane itself and not of the actin cortex.

Formally, $$E_{\textrm{el}}^{(1)}$$ reads

11 $$\begin{aligned} E_{\textrm{el}}^{(1)} = \frac{1}{2} k_c {{\int _{{\mathbb {T}}_1}}}\left( \vert \partial _s X\vert -1\right) ^2 ds, \end{aligned}$$

where $k_c$ is the mechanical parameter representing the cell membrane stretchability.

This choice is motivated by some reasoning at the discrete scale. Indeed, we consider the following very simple model of the cortex: we think of the cortex as a (closed) chain of *n* individual point masses $(X_i)_{1 \le i \le n}$, each of mass *M*/*n*. They are linked by Hookean springs of stiffness ${\tilde{k}}_c$ and of equilibrium length $\ell _0$, so that the potential energy of the spring between $X_i$ and $X_{i+1}$ has the expression

$$\begin{aligned} \frac{1}{2} {\tilde{k}}_c (\vert X_{i+1} - X_{i}\vert - \ell _0)^2. \end{aligned}$$

The total potential energy is obtained by summation over *i*:

$$\begin{aligned} \sum _i \frac{1}{2} {\tilde{k}}_c (\vert X_{i+1} - X_{i}\vert - \ell _0)^2. \end{aligned}$$

By considering the scaling ${\tilde{k}}_c = k_c \ell _0^{-2}$ with $\ell _0 \propto n^{-1}$, we can take the (formal) limit as $n \rightarrow \infty$ to recover $E_\textrm{el}^{(1)}$. Since the index *i* counts the “mass”, so does the continuous variable *s*, as explained at the beginning of Sect. 2.1, it is possible to obtain Eq. (11). One can also write $E_\textrm{el}^{(1)}$ in terms of the arc length:

$$\begin{aligned} E_{\textrm{el}}^{(1)}&= \frac{1}{2} k_c \int _0^L (\vert \partial _s X\vert - 1)^2 \vert \partial _s X\vert ^{-1} \; d\ell \ = \frac{1}{2} k_c \int _0^L ( \rho ^{-1} - 1)^2 \rho \; d\ell \,, \end{aligned}$$

so that the local energy density is convex, with its minimum in $\rho \equiv 1$, and becomes large for small or large values of $\rho$ from the reference density, i.e. either $\rho \ll 1$ or $\rho \gg 1$. We remark that, even though displacement does not appear explicitly, the contribution of $E_{\textrm{el}}^{(1)}$ tends to keep $\vert \partial _s X\vert$ (or equivalently, $\rho$), and thus the length *L*, close to one, thanks to Eq. (2). If one assumes that the actin cortex is continuously laid out (i.e. polymerized) with a density $\rho = 1$, $\rho -1$ can then be interpreted as some form of displacement, which explains the form of the right-hand side in the equation above. The factor $\rho$ can be understood as follows: for a given displacement, the local energy density grows linearly with the density of actin filaments. The above expression is similar to that of the standard potential energy for an elastic ring, except for the factor $\vert \partial _s X\vert {^{-1}} = \rho$. In other words, the local energy density is also proportional to the actin density $\rho$, which is consistent with the discrete model: for fixed *n*, as the point masses $X_i$ get far from one another, the number of springs in a given length interval decreases, and so does the force they exert.

Furthermore, we include an elastic constraint on the cell area $\left| \Omega \right|$ (which would correspond to the volume in three-dimensions). In particular, according to previous works (e.g. Kuchnir Fygenson et al. 1997), we assume that the elastic energy of the cell is minimised if the cell area is equal to a given target area $A_c^*$, i.e.

12 $$\begin{aligned} E_{\textrm{el}}^{(2)} = \mu _c \left( \left| \Omega \right| - A_c^* \right) ^2= \mu _c \left( -\frac{1}{2}{{\int _{{\mathbb {T}}_1}}}X\cdot \partial _s X^\perp \, \textrm{d}s - A_c^* \right) ^2, \end{aligned}$$

where $\mu _c$ is the mechanical parameter representing the elastic resistance of the cell bulk to variations of its area, given by the measure of the domain $\Omega$, $\left| \Omega \right|$. Physically, this models the resistance to compression of the organelles in the cytoplasm.

Concerning the term $E_{\textrm{p}}$, because of osmotic effects, we suppose that the cell is subject to an internal cytoplasmic pressure, which results in a force in the direction of the normal to the curve. The force intensity per unit length is assumed to be uniform in space and constant in time, so that the associated energy $E_{\textrm{p}}$ is

$$\begin{aligned} E_{\textrm{p}}= -p \left| \Omega \right| = \frac{p}{2} {{\int _{{\mathbb {T}}_1}}}X\cdot \partial _s X^\perp \, \textrm{d}s, \end{aligned}$$

where $p>0$ is the constant excess of pressure inside the cell, with respect to the extracellular pressure.

### Evolution of the Microtubules’ Structure and the Centrosome

As depicted in Sect. 2.1, microtubules (MTs) and the associated centrosome are the principal coupling mechanisms between the cortex and the nucleus. Microtubules are known to generate forces to position and shape the cellular organelles, and in particular the nucleus (Mofrad 2009). A number of observations (Soheilypour et al. 2015; Mofrad 2009; Stamenović et al. 2002) suggest that among all cytoskeletal components, MTs play a critical role in carrying compressive loads, behaving as passive compression-supporting elements that maintain cell shape. However, when MTs are anchored to the cell cortex, through dynein motor proteins, they are able to generate pushing forces over the cell membrane during their assembly process, and pulling forces during their disassembly process (Laan et al. 2008). Since the positioning of the nucleus and the centrosome has been found to principally depend on MTs pushing/pulling forces (Laan et al. 2008), we are here interested in giving a simplified mathematical description of such a force, directed along the microtubule axis.

In the mathematical setting put forward in this work, at every instant of time, a continuous structure of MTs, homogeneously distributed around the centrosome, located in $X_c(t)$, connects the centrosome to the points on the cortex $s_{MT}(t,\theta )= \Pi _{MT}(t,\theta )$, $\forall \theta \in [0, 2\pi )$ where MTs can anchor (see the bold line in Fig. 1).

The microtubule push/pulling force $F_{MT} (t, \theta )$ is, thus, directed along the line segment $[X_c(t), X(t, s_{MT}(\theta ))]$, that represents the microtubule (see the reference purple segments in Fig. 1). Many works in the literature focus on MT mechanical response to bending (Mizushima-Sugano et al. 1983) and radial indentation (Schaap et al. 2006), but less is known on their response to elongation. However, some works highlight interesting behaviour of the MTs with respect to forces directed along the MTs major axis (see Laan et al. 2008; Soheilypour et al. 2015), which is responsible for the positioning of the nucleus and the centrosome. In particular, in Laan et al. (2008) it was assumed that the in-line force exerted by a MT is related to its polymerizing activity, whereas in Soheilypour et al. (2015) the buckling of long MT bundles was observed, which can strongly reduce the exerted tension. In this work, we consider that the modulus of the MT force, directed along the MT major axis, is a function of the microtubule length $\left| X_c(t) - X(t, s_{MT}(\theta ))\right|$, to be specified, i.e.

13 $$\begin{aligned} F_{MT} (t, \theta ) = - f^*_{MT}\left( \left| X(t, s_{MT}(t,\theta ))- X_c(t) \right| \right) \textrm{e}_{\theta } , \end{aligned}$$

were $\textrm{e}_{\theta }$ is the outward unit vector representing the direction of a MT. In the following, we will consider $f^*_{MT}= k_{MT}^* \left| X(t, s_{MT}(t,\theta ))- X_c(t) \right| ^{-2}$, so that smaller MTs, that can polymerize the most, exert the higher forces, whereas the force decreases with the MT length, due to buckling instability. We observe that, since $f^*_{MT}>0$, the force acting on the MT is always directed along $-\textrm{e}_{\theta }$, whereas the force exerted by each MT on the cell cortex is pushing against it, being directed along $\textrm{e}_{\theta }$. We remark that the microtubule force acts on the portion of the cell cortex where $\rho _{MT} \ne 0$ [see Eq. (8)] and on the centrosome, where MTs originate. The total resultant of all microtubule forces acting on the centrosome is then,

14 $$\begin{aligned} {\bar{F}}_{MT}(t)= \int _{0}^{2\pi }F_{MT} (t, \theta ) \, \textrm{d}\theta . \end{aligned}$$

In addition to the in-line forces due to elongation, the cortex endpoints of the microtubules are also subject to a friction force, caused by the sliding of the binding complexes on the cortex. This force is directed opposite to the velocity of the centrosome relative to the cortex. The friction also generates a torque, with angular velocity $\omega$. The motion of the MT structure with respect to the angular velocity can be regarded as rigid in the sense that all MTs rotate with the same angular velocity. However, since the MT structure does not simply rotate but also resizes according to the cell shape and the microtubule organising centre location, it is not a standard rigid body rotation. As already pointed out, this is of course an approximation of the far more complex biological reality.

Defining $v(\partial _t X_c, \omega , \theta )$ the speed of the centrosome relative to the cortex endpoint of the microtubule of angle $\theta$, we have

15 $$\begin{aligned}{} & {} v(t, \partial _t X_c, \omega , \theta ) :=\,\partial _t X_c(t) - \left[ \partial _t X(t, s_{MT}(t, \theta )) - \vert X_c(t) - X(t, s_{MT}(t, \theta ))\vert \omega \textrm{e}_{\theta }^\perp \right] .\nonumber \\ \end{aligned}$$

The first term on the r.h.s of Eq. (15) represents the velocity of the centrosome, whereas the second term, between square brackets, stands for the velocity of the cortex point where the microtubule is attached. Since the MT structure is redrawn in the cell at each instant of time, without explicitly tracking the motion, elongation and/or breakage of each single MT, in order to properly define the velocity of the MT on the cortex, we have to consider the anchor point of the MT onto the cell cortex whose position is well defined and can be differentiated in time, instead of the end of the MT, which is not explicitly tracked. We remark that we consider $\partial _t X(t, s_{MT}(t, \theta ))$ as opposed to the total derivative $\frac{D}{Dt} X(t, s_{MT}(t, \theta ))$, since we want to describe the relative velocity of the centrosome with respect to the cell membrane and not with respect to the laboratory frame. Furthermore, the third term on the r.h.s. of Eq. (15) corresponds to the sliding due to the rotation of the MT structure at each instant of time, which is obtained considering the relation between the linear and the angular velocity.

For the benefit of subsequent computations, the relative velocity *v* can be also decomposed along the directions $\tau$ and $\textrm{e}_\theta$:

$$\begin{aligned} v = v_\tau \tau + v_{\textrm{e}_\theta } \textrm{e}_\theta = (\varPi _\tau + \varPi _{\textrm{e}_\theta }) v, \end{aligned}$$

where $\Pi _\tau$ and $\Pi _{e_\theta }$ are the corresponding *oblique* projections:

$$\begin{aligned} \varPi _\tau = \frac{\tau \otimes \textrm{e}_\theta ^\perp }{\tau \cdot \textrm{e}_\theta ^\perp }, \text { and } \varPi _{\textrm{e}_\theta } = \frac{\textrm{e}_\theta \otimes \tau ^\perp }{\textrm{e}_\theta \cdot \tau ^\perp }. \end{aligned}$$

Locally on the cortex, the friction of the microtubule against the cortex generates a force

$$\begin{aligned} F_\textrm{fric}= -(k_\tau \varPi _\tau + k_{\textrm{e}_\theta } \varPi _{\textrm{e}_\theta }) v, \end{aligned}$$

where $k_\tau$ and $k_{\textrm{e}_\theta }$ are the friction coefficients in the corresponding directions. In the following, for the sake of simplicity, we will take $k_{\textrm{e}_\theta } = k_\tau$, but the model can be easily generalised to the case $k_{\textrm{e}_\theta } \ne k_\tau$.

By writing $F_\textrm{int} {:}{=} {\bar{F}}_{X_c}+{\bar{F}}_{MT}$ the sum of forces acting on the microtubule structure, which include ${\bar{F}}_{MT}$ (the forces directed alongs the MTs), given by Eq. (14), and ${\bar{F}}_{X_c}$, the forces acting on the centrosome itself (that will be detailed in Sect. 2.4, see Eq. (27)), we have the following force balance in ${\mathbb {R}}^2$:

16 $$\begin{aligned} -k_\tau \left[ 2\pi \partial _t X_c + \int \left( \omega \vert X_c - X_\theta \vert \textrm{e}_\theta ^\perp - \partial _t X_\theta \right) \textrm{d}\theta \right] + F_\textrm{int} = 0, \end{aligned}$$

where we used the shorthand $X_\theta = X(s_{MT}(\theta ))$.

Since the microtubule structure has zero moment of inertia w.r.t. $X_c$, we also have the following balance of torques

$$\begin{aligned} \int (X_\theta - X_c) \times F_\textrm{fric} \;\textrm{d}\theta = 0&\Leftrightarrow -k_\tau \int (X_\theta - X_c)^\perp \cdot v\; \textrm{d}\theta = 0\\&\Leftrightarrow -k_\tau \int \vert X_\theta - X_c\vert \textrm{e}_\theta ^\perp \cdot v\; \textrm{d}\theta = 0\,, \end{aligned}$$

which can be rewritten by decomposing *v* on $\textrm{e}_\theta$ and $\textrm{e}_\theta ^\perp$:

17 $$\begin{aligned} 0&= -k_\tau \int \vert X_\theta - X_c\vert \left[ \partial _t (X_c - X_\theta )\cdot \textrm{e}_\theta ^\perp + \omega \vert X_\theta - X_c\vert \textrm{e}_\theta ^\perp \right] \textrm{d}\theta \nonumber \\&= -k_\tau \Big [\omega \int \vert X_c - X_\theta \vert ^2 + \partial _t X_c \cdot \int \vert X_c - X_\theta \vert \textrm{e}_\theta ^\perp \\&\qquad \quad - \int \vert X_c - X_\theta \vert \partial _t X_\theta \cdot \textrm{e}_\theta ^\perp \Big ] \textrm{d}\theta \,.\nonumber \end{aligned}$$

Gathering (16) and (17), we get the system

18 $$\begin{aligned} A \begin{pmatrix} \partial _t X_c \\ \omega \end{pmatrix} = B, \end{aligned}$$

where

$$\begin{aligned} A&= k_\tau \begin{pmatrix} 2 \pi I_2 &{}\quad \int \vert X_c - X_\theta \vert \textrm{e}_\theta ^\perp \\ \left( \int \vert X_c - X_\theta \vert \textrm{e}_\theta ^\perp \right) ^T &{}\quad \int \vert X_c - X_\theta \vert ^2 \end{pmatrix}\,, \quad \\ B&= \begin{pmatrix} k_\tau \int \partial _t X_\theta + F_\textrm{int} \\ k_\tau \int \vert X_c - X_\theta \vert \textrm{e}_\theta ^\perp \cdot \partial _t X_\theta \end{pmatrix}\,, \end{aligned}$$

where $I_2$ is the identity matrix in 2D. We remark that, by Jensen’s inequality^1^, we have

$$\begin{aligned} \det A = 2\pi \, k_\tau ^3 \left( 2\pi \int \vert X_c - X_\theta \vert ^2 - \left| \int \vert X_c - X_\theta \vert \textrm{e}_\theta ^\perp \right| ^2\right) = 2\pi \, k_\tau ^3\, \Delta \ge 0, \end{aligned}$$

The equality occurs only if $X_\theta$ reduces to a single point, so we can assume that the determinant is always positive. Then, system (18) can be solved to get the angular velocity $\omega$ of the MT structure and the velocity of the centrosome:

19 $$\begin{aligned} \omega&= \Delta ^{-1} \Bigg [-\left( k_\tau ^{-1} F_\textrm{int} + \int \partial _t X_\theta \right) \cdot \int \vert X_c - X_\theta \vert \textrm{e}_\theta ^\perp \nonumber \\&\qquad \qquad + 2\pi \int \vert X_c - X_\theta \vert \textrm{e}_\theta ^\perp \cdot \partial _t X_\theta \Bigg ] \end{aligned}$$

20 $$\begin{aligned} 2\pi \partial _t X_c&= - \omega \int \vert X_c - X_\theta \vert \textrm{e}_\theta ^\perp + \int \partial _t X_\theta + k_\tau ^{-1} F_\textrm{int}\,. \end{aligned}$$

### Evolution of the Nuclear Membrane

We consider the nucleus material to be harder to deform than the rest of the cell and with a negligible relaxation time. The nuclear membrane has a certain mechanical behaviour and, as previously stated, it is connected to the centrosome, which contributes to the positioning of the nucleus inside the cell. Furthermore, the nucleus cannot cross the cellular membrane and therefore interacts with it through a contact force $F_{\textrm{cont},n}$. Then, calling $F_{n}$ the forces internal to the nuclear membrane, $F_{X_cN}$ the force acting on each point of the nuclear membrane due the interaction between the nucleus and the centrosome, $F_{\textrm{cont}, n}(\sigma )$ the contact force with the cortex, the Newton’s Second Law for the nuclear membrane, neglecting inertial terms, reads

21 $$\begin{aligned} F_{n} - F_{X_cN} +F_{\textrm{cont},n} = h_v \, \partial _t Y \,. \end{aligned}$$

The meaning and derivation of the different terms on the l.h.s of Eq. (21) is depicted below, whereas the term on the r.h.s. describe the friction force acting on the nucleus. In the following, we will set the coefficient $h_v$ equal to unity, assuming that the viscous coefficient representing the interactions between the nuclear membrane and the cytosol is the same as the one representing the interaction between the cell cortex and the intra- and extra-cellular fluid. This assumption seems reasonable when the cell migrate in an adhesion-independent manner, whilst in adhesion-mediated migration the friction term in the cortex equation should also take into account the formation and breakages of adhesion points with the surrounding environment and it is certainly different from the viscous coefficient representing the interactions between a cellular/nuclear membrane and a fluid. For what concerns the l.h.s of Eq. (21), the force $F_{n}$ related to the mechanical behaviour of the membrane can be derived from the nuclear membrane energy, $E_n$, through the relation

$$\begin{aligned}F_{n}=- \vert \partial _\sigma Y\vert ^{-1} \delta E_{{n}}/ \delta Y, \end{aligned}$$

where the energy $E_n$ is the sum of all energies related to the constitutive mechanical behaviour of the nuclear envelope, i.e.

22 $$\begin{aligned} E_n = \dfrac{k_b}{2} {\int _{\Gamma _n}}(K-K_0)^2 \,\textrm{d}\ell + \lambda {\int _{\Gamma _n}}\textrm{d}\ell + \Delta p_n {\int _{\Omega _n}}\,\textrm{d}A \, + \, \mu _n \left( {\int _{\Omega _n}}\,\textrm{d}A - A_n^* \right) ^2 \,, \end{aligned}$$

where $$\textrm{d}\ell = \vert \partial _\sigma Y\vert \textrm{d}\sigma $$ is the arc length element and $$\textrm{d}A $$ the area element. The first term in Eq. (22) represents the energy associated to the membrane bending, $$k_b$$ is the bending modulus of the nuclear membrane, *K* the local curvature and $$K_0$$ the characteristic (or spontaneous) curvature, which is assumed to be zero in what follows, according to Kaoui et al. (2008). The second term represents the tensile stress acting on the membrane and $$\lambda $$ can be thought as the nucleus surface tension. $$\Delta p_n$$ is the difference between the pressure in the cytosol and the pressure inside the nucleus. Finally, the last term represents the volumetric elastic constraint associated to changes in nucleus area $$A_n$$ relative to a defined target area $$ A_{n}^*$$ and it has the same form as the constraint introduced for the whole cell in Eq. (12).

Taking all these contributions into account, the computation of the variation $\delta E_n$ of the nuclear membrane energy function (see Section A) leads to the following nuclear membrane mechanical force

$$\begin{aligned} F_n = k_b \left( \dfrac{\partial ^2 K}{\partial \ell ^2} + \dfrac{1}{2} K^3 \right) N - \lambda K \, N - \Delta p_n N - \mu _n \left( {\int _{{\mathbb {T}}_1}} Y \cdot \partial _{\sigma } Y ^\perp \, \textrm{d}\sigma -A_{n}^* \right) N \, , \end{aligned}$$

where *N* is the outward normal to the nucleus (see Fig. 1). Then, to derive a mathematical expression for the second term on the r.h.s of Eq. (21), we assume that the centrosome is linked to the nuclear membrane points thanks to cytoskeletal filaments, namely microtubules and intermediate filaments (Cooper 2000). Intermediate filaments form an intricate ring network that surrounds the nucleus of most cells and extends in the cytoplasm, where they associate with the other elements of the cytoskeleton, such as microtubules (Cooper 2000). Intermediate filaments attached to the nuclear envelope, along with microtubules, serve to position and anchor the nucleus within the cell, to redistribute forces acting on the nucleus and to provide a scaffold that integrates the components of the cytoskeleton (Cooper 2000). Therefore, assuming that each of these cytoskeletal coupled structures behaves as a sort of spring and that the network formed by intermediate filaments surrounding the nucleus leads to a uniform distribution, along the length of the nuclear membrane, of the forces coupling the centrosome to the nuclear envelope, we have

23 $$\begin{aligned} F_{X_cN} = - \dfrac{{\int _{{\mathbb {T}}_1}} k_e (X_c - Y ) \left| \partial _{\sigma } Y(\sigma )\right| \, \textrm{d}\sigma }{L_n(t)} \,, \end{aligned}$$

where $k_e$ is the elasticity of each virtual link between the centrosome and the nuclear membrane point, and $L_n$ is the length of the nuclear membrane at time *t*, which is given by

24 $$\begin{aligned} L_n(t) = {\int _{{\mathbb {T}}_1}}\left| \partial _\sigma Y(t, \sigma )\right| \, \textrm{d}\sigma \,. \end{aligned}$$

Since the 2D setting that we are considering is to be interpreted as the projection of the 3D geometry, we assume that the rest length for the connection between the nucleus centroid and the centrosome is equal to zero. This condition represents the 3D situation in which the centrosome is on the top or bottom of the nucleus. It is placed at the centre of the 2D projection of the nucleus. If one assumes that $k_e$ is constant, it is possible to rewrite (23) as

25 $$\begin{aligned} F_{X_cN} =- k_e (X_c -{\bar{Y}}) \,, \end{aligned}$$

where ${\bar{Y}}$ is the centroid of the nucleus (see Fig. 1) defined as

26 $$\begin{aligned} {\bar{Y}}(t)= \dfrac{1}{L_n} {\int _{{\mathbb {T}}_1}} Y(t, \sigma ) \left| \partial _{\sigma } Y(t, \sigma )\right| \, \textrm{d}\sigma . \end{aligned}$$

Under the same assumption, it is also possible to define the total force ${\bar{F}}_{X_c}$ acting on the centrosome due to all centrosome-nucleus interactions, which is thus part of $F_\textrm{int}$ in Eq. (16):

27 $$\begin{aligned} {\bar{F}}_{X_c} = - {\int _{{\mathbb {T}}_1}} k_e (X_c - Y ) \left| \partial _{\sigma } Y(\sigma )\right| \, \textrm{d}\sigma = - L_n k_e (X_c -{\bar{Y}}) \,. \end{aligned}$$

We observe that the presence of an elastic coupling between the nucleus centroid and the centrosome, specified in Eqs. (25) and (27), is in agreement with the data-driven theoretical approach proposed in Brückner et al. (2022). Specifically, Brückner et al. (2022) develop a model for protrusion and polarity dynamics in confined cell migration, combining experimental data inference with a mechanistic approach, based on an elastic coupling between the cell nucleus centroid and the center of the cytoplasm protrusion, combined with a non-specific friction acting both on the cell and the nucleus and a proper description of cell polarity.

Finally, as previously mentioned, the nucleus is constrained to remain within the cell, since it cannot cross the cortex. This is guaranteed by including a penalization, represented by the last term on the l.h.s of Eq. (21). The contact force is given by

28 $$\begin{aligned} \begin{aligned} F_{\textrm{cont},n}(\sigma )&= -\nabla _x\left[ \int _{{\mathbb {T}}^1} W_\textrm{cont}(\vert x - X(s)\vert ) \;ds\right] \bigg \vert _{x=Y(\sigma )} \\&= -\int \frac{Y(\sigma ) - X(s)}{\vert Y(\sigma ) - X(s)\vert }W'_\textrm{cont}(\vert Y(\sigma ) - X(s)\vert ) \;ds. \end{aligned} \end{aligned}$$

where $W_\textrm{cont}$ is a decreasing function with compact support.

By symmetry, we specify the corresponding force acting on the cortex, $F_\textrm{cont}$, appearing in (8).

29 $$\begin{aligned} F_\textrm{cont}(s) = \int \frac{Y(\sigma ) - X(s)}{\vert Y(\sigma ) - X(s)\vert }W'_\textrm{cont}(\vert Y(\sigma ) - X(s)\vert ) \;d\sigma , \end{aligned}$$

so that $$\int F_\textrm{cont}(s) \; ds = - \int F_{\textrm{cont},n}(\sigma ) \; d\sigma \,.$$

We remark that the inclusion of the contact constraint is essential in order to prevent the penetration of the nucleus and the cell cortex, for any range of the model parameters and channel size. Indeed, the inclusion of the MTs force given by Eq. (13) is not enough, since it does not directly act on the nuclear membrane points, but its resultant (see Eq. (14)) is applied to the centrosome. The location of the centrosome, in turns, affects the positioning of the center of mass of the nucleus, but without any contact force between the nucleus and the cell cortex, the penetration between some regions of the nucleus and the cortex could take place. The specific functional form used for $W_\textrm{cont}$ will be discussed in Sect. 4.

## Numerical Discretisation

The whole system composed by Eqs. (8)–(18)–(21) is discretised in space using finite differences, the resulting system of ODEs is then solved using split step time stepping scheme of order one. At each time step, the new position of the nuclear membrane is computed using the explicit scheme described below. Then, the position of the cortex and centrosome are updated using a semi-implicit scheme.

### Cortex, Centrosome and Microtubules Structure

We assume the total mass of actin on the cortex to be normalised to 1 and we introduce $N_1\in {\mathbb {N}}$ grid-points for the discretisation of *s* (corresponding to the cortex) such that $s_i = i\Delta s$, $\Delta s =1/N_1$ for $i \in \{0, N_1 - 1\}$. Given a time step $\Delta t>0$, we denote $t^j = j\Delta t$. In what follows, subscripts (resp. subscripts) correspond to space (resp. time), and we define $X_i^j{:}{=}X(t^j, s_i)$. For the sake of legibility, indices or exponents are omitted if possible. For integrals, we use the midpoint rule, meaning that

$$\begin{aligned} {\int _{{\mathbb {T}}_1}} f(X(s)) \; ds \simeq \Delta s \sum _{0 \le i < N_1} f(X_i), \end{aligned}$$

and

$$\begin{aligned} \int f(X(s(\ell ))) \; d \ell \simeq \sum _{0 \le i < N_1} f(X_i) \vert X_{i+1} - X_{i-1}\vert /2. \end{aligned}$$

We define $\tau _i = \frac{X_{i+1} - X_{i}}{\vert X_{i+1} - X_{i}\vert }$ and $n_i = \tau _i^\perp$.

The cortex elastic force, the bulk elasticity and pressure forces are discretised as

30 $$\begin{aligned} F_{c,i}^j= & {} \frac{k_c}{\Delta s} \left( \left( \frac{\vert X_{i+1}^j - X_i^j\vert }{\Delta s} - 1\right) \tau _i^j - \left( \frac{\vert X_{i}^j - X_{i-1}^j\vert }{\Delta s} - 1\right) \tau _{i-1}^j \right) \nonumber \\{} & {} \quad + \left( p - \mu _c (A^j - A_c^*)\right) \frac{X_{i+1}^j - X_{i-1}^j}{2\Delta s}, \end{aligned}$$

where the (polygonal) area $A^j$ is computed as

$$\begin{aligned} A^j = \sum _i \frac{1}{4} X_i^j \cdot (\vert X_{i+1}^j - X_i^j\vert n_i^j + \vert X_{i}^j - X_{i-1}^j\vert n_{i-1}^j). \end{aligned}$$

The interaction force with the wall simply becomes

$$\begin{aligned} F_{\textrm{wall},i} = -\nabla W_\textrm{wall}(X_i), \end{aligned}$$

whereas the transport term in (9) reads

$$\begin{aligned} F_{T,i}^j = \tau _i^j \Delta s\sum _{k\le i} f_i^k. \end{aligned}$$

Following (10), the compensating force $F_{\textrm{comp}}$ must satisfy

$$\begin{aligned} \sum _{0 \le i< N_1} F_{\textrm{comp},i} = -\sum _{0 \le i < N_1} X_i f_i \frac{\vert X_{i+1} - X_{i-1}\vert }{2}. \end{aligned}$$

In the present work, we are mostly interested in the impact of the nucleus on the dynamics, so we can choose $F_{\textrm{comp},i}$ independent of *i* for simplicity.

The computation of $\Pi _{MT}$ (or equivalently $s_{MT}(\theta )$) and $\rho _{MT,i}^j$ requires the construction of the visibility polygon of the cortex from the centrosome. We do not discuss the construction here and refer to Lee (1983) and Joe and Simpson (1987) for details. The quadrature formulae and corresponding expression for $\Pi _{MT}$ are detailed in “Appendix B”.

It remains to deal with the contact force between the cell cortex and the nuclear membrane, which we simply take as the discretisation of (29):

$$\begin{aligned} F_{\textrm{cont},i} = \sum _k \frac{Y_k - X_i}{\vert Y_k - X_i\vert } W_\textrm{cont}'(\vert Y_k - X_i\vert ). \end{aligned}$$

The Eq. (18) for the angular velocity $\omega$ of the MT structure and the velocity of the centrosome is discretised similarly in time and space, using the quadrature formulae reported in the Appendix (see section B) to compute the integral of the different quantities.

For the time iteration, we use an implicit Euler scheme, so that for both the cortex and the centrosome we obtain a system of the following form at each time step:

$$\begin{aligned} \left[ \begin{pmatrix} I_{2N_1} &{} &{} \\ &{} I_{2} &{} \\ &{} &{} 0 \end{pmatrix} + \Delta t \begin{pmatrix} M_{X,X} &{} M_{X_c,X}^T &{} M_{\omega ,X}^T \\ M_{X_c,X} &{} M_{X_c,X_c} &{} M_{X_c,\omega } \\ M_{\omega ,X} &{} M_{\omega ,X_c} &{} M_{\omega ,\omega } \end{pmatrix} \right] \begin{pmatrix} \vdots \\ X_{i,1}^{j+1} - X_{i,1}^j \\ \vdots \\ X_{c}^{j+1} - X_{c}^j \\ \omega ^{j+1} - \omega ^j \end{pmatrix} = \Delta t \begin{pmatrix} \vdots \\ r_{i}^{j} \\ \vdots \\ r_{c}^{j} \\ r_\omega ^j \end{pmatrix}, \end{aligned}$$

where the $r_i, r_c, r_\omega$ on the r.h.s. correspond to the discretized terms detailed in this section, and the matrices *M* are the corresponding jacobian matrices. The left-most matrix above corresponds to the discretization of the time derivatives. Since the problem is formulated in term of the angular velocity $\omega$, whose governing equation do not involve derivatives in time, the last element of the diagonal is zero.

### Nuclear Membrane

The evolution of the nuclear membrane is derived rewriting the proposed equation (21) with the formulation and the efficient discretisation scheme proposed in Mikula and Ševčovič (2004) and Beneš et al. (2009) appropriately adapted to our setting. The method basically consists in splitting the velocity guiding the evolution of the nuclear membrane, $\partial _t Y$, into its normal component, $\beta$, and its tangential one, $\alpha$, so that $\partial _t Y= \beta N + \alpha T$. The normal velocity is the one determining the variations in the nuclear morphology, whereas the tangential component has no impact on the shape of the evolving curve, but it governs the distribution of the nodes along the nuclear envelope. The evolution of the nucleus is then given by the following system (see the Appendix, Sect. C.1 and C.2, for further details)

31 $$\begin{aligned} {\left\{ \begin{array}{ll} \partial _t K &{}= - k_b \left( \partial _\ell ^4 K + \frac{1}{2} \partial _\ell ^2(K^3)\right) + \partial _\ell (\alpha K) - K(K\beta + \partial _\ell \alpha ) \\ {} &{}\quad + \partial _\ell ^2 (\nabla W(Y(\ell )) \cdot N) + \partial _\ell ^2 (W(Y(\ell )) K) \\ \partial _t \nu &{}= -k_b \partial _\ell ^4 \nu - \frac{k_b}{2}\partial _\ell (\partial _\ell \nu )^3 + \partial _\ell (\nabla W(Y(\ell ))\cdot N) \\ &{}\quad + \partial _\ell (W \partial _\ell \nu ) + \alpha \partial _\ell \nu \ \\ \partial _t \eta &{}= K \beta + \partial _\ell \alpha \\ \partial _t Y &{}= \alpha \partial _\ell Y - k_b \left( \partial _\ell ^4 Y + \frac{3}{2} \partial _\ell (K^2 \partial _\ell Y)\right) + W(Y(\ell )) \partial _\ell ^2 Y \\ {} &{} - \Delta p_n \, N - \mu _n (A_n -A_{n}^*) N - \left( \nabla W(Y(\ell )) \cdot N \right) N , \end{array}\right. } \end{aligned}$$

where $\ell$ is the arc length, *K*, $\nu$ and $\eta = \log \vert \partial _\sigma Y\vert$ are, respectively, the curvature, the tangential angle and the logarithm of the local length of the nucleus envelope $\Gamma _n$ at a point $Y \in \Gamma _n$, whereas $W(Y(\ell ))$ is the potential combining the contact interaction with the cell cortex, the elastic constraint with the centrosome and the nuclear membrane surface tension. In Eq. (31) we take

32 $$\begin{aligned} \partial _\ell \alpha&= -K\beta + \langle K \beta \rangle + (L_n/g-1) \zeta \end{aligned}$$

33 $$\begin{aligned} \beta&= k_b \left( \partial _\ell ^2 K + \frac{1}{2} K^3\right) - \Delta p_n - \mu _n (A_n -A_{n}^*) - \nabla W \cdot N - W K \,, \end{aligned}$$

where $g = |\partial _\sigma Y|$, $\langle u \rangle = L_n^{-1} \int _{\Gamma _n} u(\ell ) \; \textrm{d}\ell$ denotes the average of *u* over the whole curve $\Gamma _n$, which makes (32) a non-local equation, whereas $\zeta >0$ is a given positive constant, included in order to avoid nodes to concentrates on points, which would lead to poor approximation and eventually inversion of ill-conditioned matrices.

To write the corresponding discretisation of (31), we uniformly discretised the fixed parameterization interval [0, 1] in $N_2$ subintervals, each of equal length $h=1/N_2$ and indexed by $i \in \{0, N_2-1\}$. For time, we use the same discretisation introduced for the cell membrane, so that the point $Y(ih, j\Delta t)$ is written $Y_i^j$. The measure of the finite element $[Y_{i-1}^j, Y_i^j]$ at time $t_j$, is given by $r_i = \vert Y_{i-1} - Y_i\vert$. Then, the system of equations (31)–(32)–(33) is solved for the discrete quantities $\alpha _i^j$, $\beta _i^j$, $K_i^j$, $\nu _i^j$, $\eta _i^j$, $Y_i^j$. In particular $\alpha _i^j$ denotes the tangential velocity of the node $Y_i^j$, whereas $\beta _i^j$, $K_i^j$, $\nu _i^j$, $\eta _i^j$ are piecewise constant approximations of the corresponding quantities on the finite element $[Y_{i-1}^j, Y_i^j]$.

The algebraic system determining the evolution of the nuclear envelope is reported in the “Appendix C.2”, along with some comments on the derivation of the discretised equations. Finally, the discretised system of equations has been solved implementing a numerical code with Julia^2^ (Bezanson et al. 2017).

## Numerical Experiments

### Setup

**Fig. 3:**
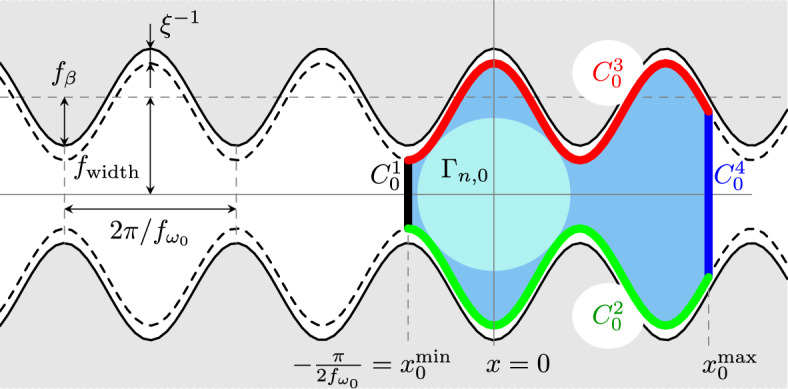
Left: The sinusoidal channels used in the numerical simulation are described by the parameters: (1) $f_\textrm{width}$, representing half of the mean width of the channel, (2) $f_{\beta }$, which is the amplitude of the oscillation of the wall, (3) $f_{\omega _0}$, which is the pulsation of the sinusoidal channel. Right: Illustration of the construction of the initial data $C_0$ and its components $C_i^0$. The value of $x_0^\textrm{min}$ is fixed to $-\pi / 2 f_{\omega _0}$, and $x_0^\textrm{max}$ is chosen so that the initial cell area (blue) is equal to $A_c^*$ (Color figure online)

In this section, we present the numerical results obtained solving the discretised system of equations presented in Sect. 3. In particular, we consider channels with structured side walls of the following form:

$$\begin{aligned} \Omega _\textrm{wall} = \left\{ (x, y) \in {\mathbb {R}}^2: \vert y\vert \le {f_{\textrm{wall}}}(x) = f_{\beta }\, \sin ( f_{\omega _0} x) + { f_\textrm{width}} \right\} , \end{aligned}$$

where $f_\textrm{width}$ represents half of the mean width of the channel, $f_{\beta } < f_\textrm{width}$ is the amplitude of the oscillation of the wall, and $f_{\omega _0}$ is the pulsation of the sinusoidal channel (see Fig. 3 for an illustration of these quantities). We remark that for $f_{\beta }=0$, one recovers the flat walls geometry. We then choose

$$\begin{aligned} F_\textrm{wall}(x, y) = \nabla \left[ g_{ \xi }({f_{\textrm{wall}}}(x) + y) + g_{ \xi }({f_{\textrm{wall}}}(x) - y) \right] \,, \end{aligned}$$

where $g_{ \xi }(x) = { -}\min ({ \xi } x - 1, 0)^2 \log ({ \xi } x)$ is a smooth barrier function with $\lim _{x \rightarrow 0^+} g_{ \xi }(x) = +\infty$ and $g_{ \xi }(x) = 0$ for $x > { \xi ^{-1}}$. Analogously, for describing the contact force between the nucleus and the cell cortex in Eqs. (28)–(29), we use $W_\textrm{cont}(r) = k_\textrm{cont} g_{\xi _\textrm{cont}}(r)$, where $k_\textrm{cont}, \xi _\textrm{cont} > 0$ are parameters.

Concerning the polymerization, we follow Sect. 2.2 and choose ${\tilde{f}}$ as a super-Gaussian: ${\tilde{f}} = \exp (-(\frac{x^2}{2w})^P)/C(t)$, where *C* is a normalisation factor. We choose $w = 0.5$ and $P = 3$.

Finally, we will consider the case in which the cell is represented

- only by the cell envelope, as done in Jankowiak et al. (2020), for comparison;
- by the cell envelope and the cell nucleus, linked together by the microtubule structure, as explained in the Sect. 2.

The initial condition for the cell cortex is chosen as the evenly spaced discretisation of a closed curve $C_0$ which matches the side walls of the channel—albeit with a smaller width—in order to have an initial cell area equal to the target area $A_c^*$. More precisely, it is the union of the following 4 curves:

- $$C_0^1 {:}{=} \{(x_0^\textrm{min}, y) \in {\mathbb {R}}^2: x_0^\textrm{min} = -\frac{\pi }{2 f_{\omega _0}}, -{f_{\textrm{wall}}}(x_0^\textrm{min}) \le y \le {f_{\textrm{wall}}}(x_0^\textrm{min})\}$$
- $$C_0^2 {:}{=} \{(x, y) \in {\mathbb {R}}^2: x_0^\textrm{min} \le x \le x_0^\textrm{max}, y = - {f_{\textrm{wall}}}(x) + {\xi ^{-1}}\}$$
- $$C_0^3 {:}{=} \{(x, y) \in {\mathbb {R}}^2: x_0^\textrm{min} \le x \le x_0^\textrm{max}, y = {f_{\textrm{wall}}}(x) - { \xi ^{-1}}\}$$
- $$C_0^4 {:}{=} \{(x_0^\textrm{max}, y) \in {\mathbb {R}}^2: -{f_{\textrm{wall}}}(x_0^\textrm{max}) \le y \le {f_{\textrm{wall}}}(x_0^\textrm{max})\}$$

where $x_0^\textrm{max}$ is chosen so that the area enclosed by $C_0$ is $A_c^*$. The initial condition for the nucleus is the circle $\Gamma _{n,0}$ centred on $(\pi /2 f_{\omega _0}, 0)$ and such that $\Gamma _{n,0} + B_{{ \xi }^{-1}}(0) \subset \Omega _\textrm{wall}$, where $B_{{\xi }^{-1}}(0)$ is the open ball of radius ${ \xi }^{-1}$ and $+$ denotes the Minkowski sum. This construction is illustrated in Fig. 3 (right).

The spatio-temporal evolution of the cell and nuclear envelope for some benchmark simulations are reported in Sect. 4.2, whereas the effect of the different parameters of the model on cell ability to move and its velocity inside sinusoidal channels is investigated in Sect. 4.3.

### Results: To Move or Not to Move

In this section, we investigate the ability of the model to reproduce cell migration inside a channel and the spatio-temporal evolution of the cell and the nucleus shapes.

**Fig. 4:**
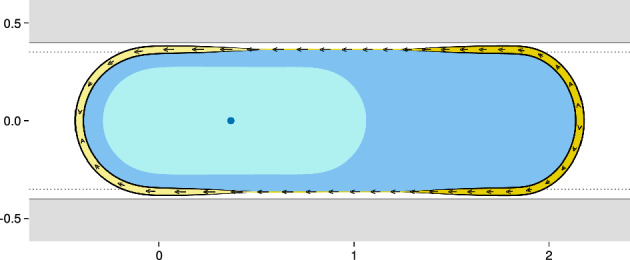
Equilibrium configuration for $f_\beta = 0$, the values for the remaining parameters are presented in Table 1. The cell is in blue and the nucleus in light blue. The dark blue dot is the centrosome. The thickness of the yellow (resp. light yellow) region on the cell membrane indicate the strength of the polymerization (resp. depolymerization). The arrows indicate the flow of the cortex relative to the cell (Color figure online)

We first consider the case of a cell positioned inside a channel with flat walls ($f_\beta = 0$): in this case, independently on the widths of the channel, no migration is observed in the simulations, in spite of polarization and corresponding cortex flow (see Fig. 4). This is due to the total balance in the transport of actin in the cell. If one only considers polymerization and the retrograde flow of actin, one expects forward movement. However, actin which is depolymerized at the back has to be transported towards the front. This mechanism, which is not modelled explicitly but taken into account through the force $F_\textrm{comp}$, balances with the actin flow in the cortex, leading to a stationary cell. If one includes additional dissipative effects, then motion can be expected again (Torres-Sánchez et al. 2019).

This result confirms that adhesion-free motility relies on structured confinement and it is in agreement with the experiments performed on leukocytes where retrograde cortical flow can be observed without motion of the cell (Reversat et al. 2020) and with the model described in Jankowiak et al. (2020), where the nucleus is not considered.

We then describe the motion of the cell inside a channel with sinusoidal walls. In this configuration, the cell takes an hourglass configuration with nucleus deformation which is often observed in vivo, so that channels of this type can be argued to mimic the inter- and extra-cellular space in which cells naturally migrate.

**Fig. 5:**
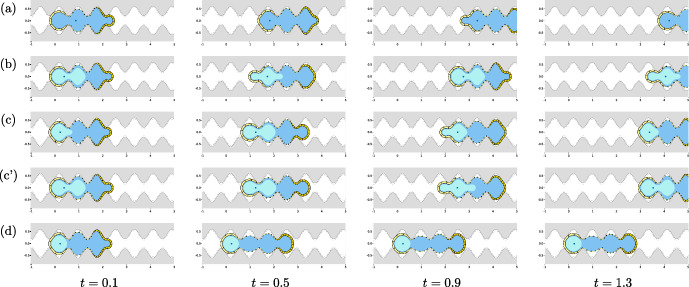
Snapshots of the cortex/nucleus/centrosome system at different times, without nucleus (**a**) and with increasing nucleus stiffness $k_b$. The average velocity decreases in the presence of the nucleus, and decreases further as $k_b$ increases [$k_b = 10^{-2.5}$ (**b**), $k_b= 10^{-1.5}$ (**c**)], eventually reaching 0 [$k_b = 10^{-0.5}$ (**d**)]. The row (**c**’) illustrates the situation for a larger value of the nucleus area constraint relaxation parameter $\mu _n = 100$. Other rows correspond to $\mu _n = 50$. The remaining parameters are presented in Table 1

In this case, as it is known from biology, the capability of the cell to migrate inside the channel and its speed of migration are highly influenced by the presence of the nucleus, which is the stiffest part in the cell and can therefore remain stuck in the rear of the cell, preventing cell motion. In the present model, the resistance of the nucleus to deformations is highly controlled by the bending modulus $k_b$ and by the elastic area-change constraint $\mu _n$. Therefore, in Fig. 5 (and in the videos in the Supplementary materials) we report the evolution of the cell and the nucleus shapes at the same instant of time, for some typical simulations, obtained varying the parameters $k_b$ and $\mu _n$. Namely, we consider

- the migrating cell without the cell nucleus, as done in Jankowiak et al. (2020) (Fig. 5a);
- the migrating cell with a low bending modulus of the nucleus (Fig. 5b, with ${k_b} = 10^{-2.5}, \mu _n = 50$);
- the migrating cell with an intermediate bending modulus of the nucleus (Fig. 5c, with ${k_b} = 10^{-1.5}, \mu _n = 50$);
- the migrating cell with a higher value of the relaxation parameter $\mu _n$, and the same bending modulus as in (c) (Fig. 5c’, with ${k_b} = 10^{-1.5}, {\mu _n} = 100$);
- the non-migrating cell with a high bending modulus of the nucleus (Fig. 5d, with $k_b = 10^{-0.5}, \mu _n = 50$).

The other dimensionless parameters chosen in the simulation are summarised in Table 1. We observe that, for the particular choice of parameters set in the simulations reported in Fig. 5a–c’, the cell is able to migrate inside the channel even when the nucleus is explicitly modelled. The cell and the nucleus size and shape change during the motion as a balance of the cell area and nucleus area penalisations, their mechanical properties, microtubules and polymerizing forces, and the contact with the channel wall. In particular, it is possible to see that the cell first protrudes the cytoplasm to fill the maximum of sinusoidal spaces ahead the cell nucleus. The maximum cytoplasm extension is controlled by the cell target area, the cell membrane target surface, and by the mechanical parameters of the cell membrane. Since these parameters are kept fixed in the simulations in Fig. 5, the cytoplasm extension inside the channel is comparable for the different cases. When the cytoplasm completely fills the new space, the nucleus is pulled by the microtutubule structure through the constriction in the channel and it forms a bleb in the front until the nucleus is pushed inside the second constriction in the sinusoidal channel. When the nucleus has a low bending stiffness ${k_b}$ (see Fig. 5b), it acquires an hourglass shape by passing through constrictions in the channel and the formation of nuclear protrusions is evident both at the cell front and at the cell rear. However, for higher values of bending stiffness (see Fig. 5c) the nucleus shrinks to pass through constrictions and the formation of blebs and the development of an hourglass shape are less pronounced. Moreover, if we increase the nucleus area-change constraint, $\mu _n$, by keeping the value of $k_b$ fixed, the nucleus cannot shrink to pass through the constrictions and intense nuclear deformations, with blebs both in the front and in the rear of the nucleus, are observed (see Fig. 5c’). In all these cases, when the nucleus fills the new sinusoidal space ahead the cell, the cytoplasm protrudes in the next available sinusoidal space and the process is repeated cyclically, allowing the cell to move forward inside the channel.

We remark that, although the cell motion occurs inside a simplified geometry, the nucleus hourglass deformation and the formation of blebs in correspondence of small openings in the extracellular space is a characteristic observed during cell motion inside intricate ECM (Wolf et al. 2007, 2013; Beadle et al. 2008).

Furthermore, the importance of including the description of the nucleus becomes clear from simulations reported in Fig. 5d, in order to account for those situations in which the cell can remain trapped inside the channel, because the nucleus cannot deform and squeeze through the small opening of the channel, thus preventing the cell motion. Indeed, in this last case, even though the cytoplasm protrudes inside the channel and the nucleus is pulled by microtubules, the energy required to deform and bend the nucleus is too high (due to the high value of $k_b$), so that the nucleus gets stuck in the rear of the cell (see Fig. 5d). This finding is in qualitative agreement with a number of experimental works, such as Wolf et al. (2007, 2013), Rolli et al. (2010) and Beadle et al. (2008), where the cell migratory capability is associated with nuclear deformations, and the existence of a critical ECM gap size below which cell migration is entirely hampered in the absence during non-proteolytic has been observed. Such a critical size was termed “the physical limit of cell migration” (Wolf et al. 2013). In particular is has been observed that, when passing through constrictions, the nucleus shape can strongly deviate from the spherical one. Specifically, the shape of the deformed nucleus inside regular cylindrical channels can be approximated either by a prolate ellipsoid (Versaevel et al. 2012; Friedl et al. 2011) or by a cigar-like shape (Friedl et al. 2011), whereas inside structured channels or complex 3D extracellular matrix, the shape of the nucleus can highly vary, from a regular hourglass shape to more irregular nuclear conformations (see the experimental pictures reported in Friedl et al. 2011; Davidson et al. 2020, 2014).

Moreover, the numerical results in our work are in agreement with other mathematical models, dealing with the influence of the mechanical properties of the nucleus on the cell’s ability to migrate in channels composed of extracellular matrix (Giverso et al. 2014, 2018; Scianna et al. 2013; Scianna and Preziosi 2013, 2014) and through a dense network of static cells (Lee et al. 2017). In particular, with respect to previous mechanical models (Giverso et al. 2014, 2018), this work provides better insights into the phenomenon, since the whole dynamics of the process is investigated, along with the influence of the cell membrane. Furthermore, the motion of the cell in this case does not require the presence of an external flux as in Lee et al. (2017), but only relies on cell deformation, cortex polymerization and the microtubule activity. Finally, compared with previous models derived using an extended version of a Cellular Potts Model (Scianna et al. 2013; Scianna and Preziosi 2013, 2014), this work allows, in principle, to obtain quantitative results of the whole migratory process, by including into the model identifiable mechanical parameters.

Of course, the dynamics of cell and nucleus deformation and motion that can be reproduced by our model is wider than the one captured by these benchmark simulations, therefore we will investigate in Sect. 4.3 the dependence of the mean cell speed and the nucleus shape on the parameters of the model.

### Influence of the Model Parameters

**Fig. 6:**
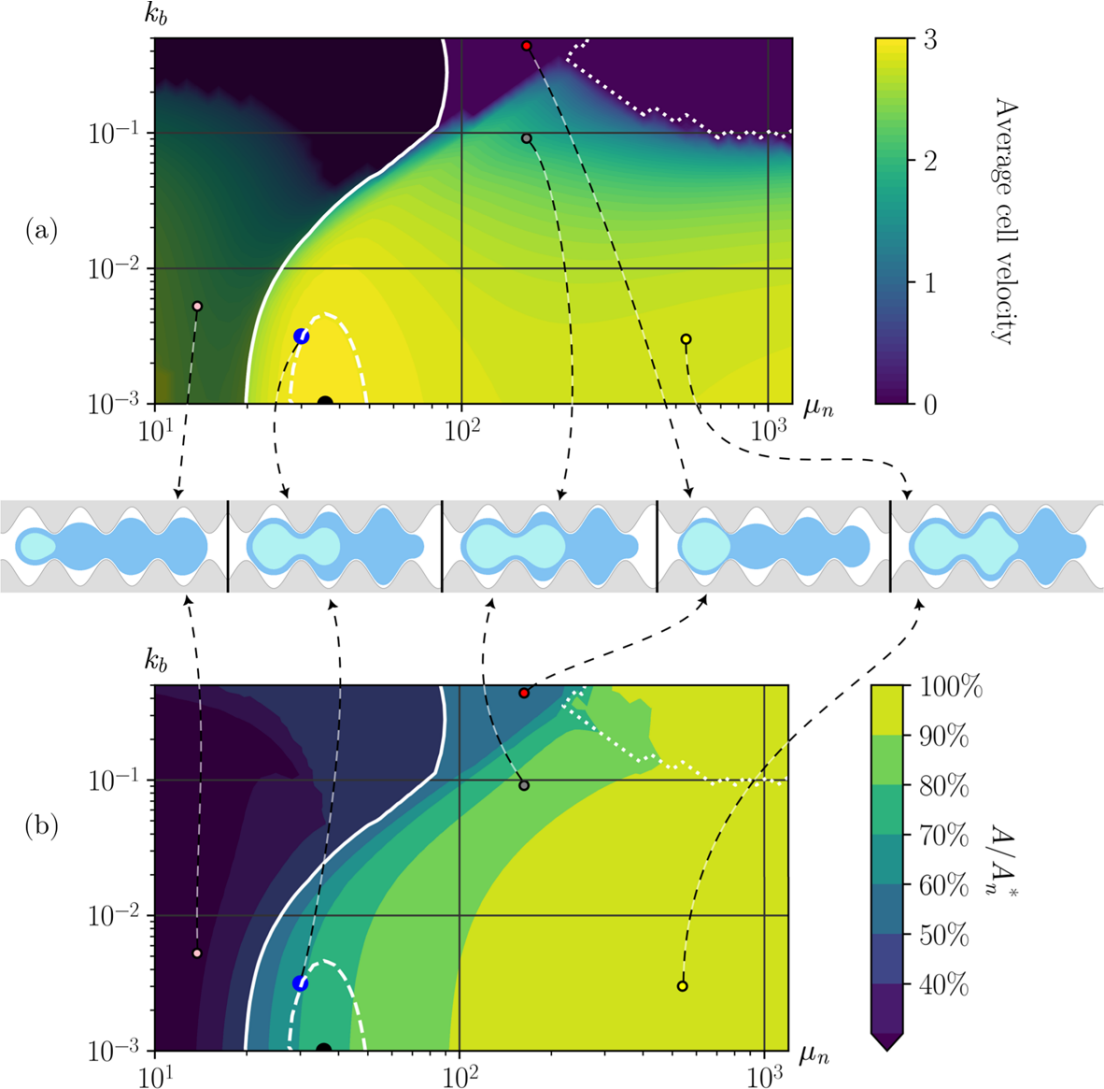
Average cell speed (**a**) and ratio between the average and the target nucleus areas (**b**) while moving inside a sinusoidal channel. The shaded region on the left corresponds to a ratio $A/A_n^* < 50\%$. The region bounded by the dotted line corresponds to $A/A_n^* > 70\%$ and zero speed. The black dot denotes the point of maximum speed, while region for which the velocity is above $99\%$ of the maximum velocity is marked with the dashed white line. The blue dot corresponds to the values of $\mu _n$ and $k_{b}$ taken for Fig. 7. For select parameters, marked by coloured dots, the cell and the nucleus are illustrated. The parameters used are given in Table 1 (Color figure online)

In order to understand how the different parameters of the model affect the capability of the cell to migrate inside sinusoidal channels and its mean speed, we perform a set of numerical experiments, changing one parameter at a time. Indeed, the model put forward in the present paper makes it hard to predict analytically the average velocity of a cell moving inside a structured channels. Therefore, we should rely on numerical simulations. To compare the results, we defined the mean cell speed as the average speed of the tip of the cell over one period of the cyclic motion, and the mean nucleus area as the average area enclosed by the nuclear membrane over the same period. The value of the mean cell speed for varying nuclear stiffness is shown in Fig. 6a, with the area-change constraint ($\mu _n$) and the bending modulus of the nucleus ($k_b$) along the horizontal and vertical axes, respectively. From Fig. 6a, one sees that the capability of the cell to move inside the channel and its speed highly depends on the nucleus mechanical properties that determine nucleus ability to deform, as it is apparent from the insets. These show the deformation of the nucleus at a given instant of time for different sets of parameters (marked with coloured dots in the parameter space).

Indeed, cell migration is hampered when the energy needed to bend the nuclear envelope is too high (i.e. for high values of the parameter $k_b$, corresponding to the red dot in the parameter space), since in this case the nucleus is not able to deform much and acquire the hourglass shape required to pass the constrictions in the sinusoidal channel and it stays round, occupying the space between two constrictions. Conversely, decreasing the value of $k_b$ (see the gray dot in Fig. 6a and the inset with the corresponding nucleus shape), and keeping the value of $\mu _n$ fixed, the nucleus can deform and pass through constrictions. Therefore, for a given value of $\mu _n$, the speed of the cell decreases for increasing values of the bending modulus $k_b$, until the cell is stuck inside the channel (zero speed). The threshold for $k_b$ allowing cell motion depends on the value of $\mu _n$.

Specifically, the constraint imposed by the bending stiffness of the nucleus is more restrictive when the chromatin inside the nucleus is little compactible, i.e. for high values of the parameter $\mu _n$. Indeed, when $\mu _n$ is sufficiently high, the nuclear deformations occur while maintaining the nuclear area close to the target one $A_n^*$. This is evident looking at the plot in Fig. 6b, where we report the average nucleus area (still computed over one period of the cyclic motion) with respect to the nucleus target area, for the same values of the nucleus mechanical parameters used in Fig. 6a: for high values of the parameter $\mu _n$ the nucleus moves preserving in average more than the $90\%$ of its target area. In this case, the deformed nucleus even pinches twice (see the deformed nucleus in correspondence of the yellow dot in Fig. 6).

The plot in Fig. 6b also allows to comment on the admissibility of the cell velocities predicted by the model. Indeed, from Fig. 6b it is clear that for small values of the parameters $\mu _n$, the nucleus can decrease its area (which would correspond to the volume in a three-dimensional simulation) below a physiological threshold (shaded region on the left of Fig. 6b). Even though there is biological evidence (Friedl et al. 2011; Rowat et al. 2006; Versaevel et al. 2012) that the volume of the nucleus is not preserved during large elongations—which suggests that the nuclear envelope is permeable to aqueous material and that the chromatin structure can compact itself (chromatin condensation)—the nucleus volume cannot shrink under a minimum threshold. In particular, in Rowat et al. (2006), it was shown that although nuclei experienced a marked loss of total volume under aspiration, it stabilized above 30–40% of the initial nuclear volume. Therefore, mechanical parameters allowing the nuclear area going below the 30–40% of the target area should be disregarded (see the white isoline in Fig. 6a, corresponding to the threshold $A/A_n^* = 50\%$). We observe that, relaxing the constraint on the area change (i.e. lowering the value of $\mu _n$), so that the nucleus can shrink, the cell can move inside the channel even for high values of bending modulus $k_b$, albeit with lower velocity (green region in the top of Fig. 6a). In this case, the nucleus maintains an elongated ellipsoidal shape and does not acquire an hourglass deformation as the cell moves inside the channel (see the nuclear shape corresponding to the white dot in Fig. 6).

Furthermore, the proposed model predicts the existence of an optimal region in the space of the mechanical parameters $k_b$-$\mu _n$ for which the cell speed is maximal (region delimited by the white dashed line in Fig. 6a, with the black dot corresponding to the maximum cell speed) and the nuclear area is in the range 70–85% of the target area.

**Table 1:** Values and ranges of the dimensionless parameters used for the numerical experiments

	Symbol	Value	Range
*Cell cortex related parameters*			
Cell/environment pressure differential	$\Delta p_c$	2.56	
Membrane elasticity	$k_c$	0.3	
Cell target area	$A_c^*$	1.8	
Cell area constraint relaxation constant	$\mu _c$	50	
Cortex polymerization rate	$r_\textrm{pol}$	10	
*Nucleus related parameters*			
Nucleus/cell pressure differential	$\Delta p_n$	1	
Nucleus target area	$A_n^*$	0.7	
Nucleus area constraint relaxation constant	$\mu _n$	30	$[10,10^3]$ $^\textrm{b}$
Nucleus bending stiffness	$k_{b}$	$10^{-2.5}$	$[10^{-3}, 5\times 10^{-1}]$ $^\textrm{b}$
			$\{10^{-2.5}, 10^{-1.5}, 10^{-0.5} \}$ $^\textrm{c}$
Nucleus/cortex interaction charact. length	$\xi _\textrm{cont}$	10	
Nucleus/cortex interaction coefficient	$k_\textrm{cont}$	5	
*Centrosome related parameters*			
Microtubules friction coefficient	$k_\tau$	$10^{-4}$	
Centrosome link stiffness	$k_{e}$	$10^{-3}$	
*Channel geometry related parameters*			
Sharpness$^\textrm{a}$	$\xi$	20	
Depth	$f_\beta$	0.2	[0.03, 0.35] $^\textrm{d}$
Pulsation	$f_{\omega _0}$	8	[5, 11.7] $^\textrm{d}$
Mean half width	$f_\textrm{width}$	0.4	[0.27, 0.8] $^\textrm{d}$
*Numerical parameters*			
Size of the cortex discretization	$N_c$	250	
Initial time step	$\Delta t$	$2 \times 10^{-4}$	
Size of the nucleus discretization	$N_n$	200	

**Fig. 7:**
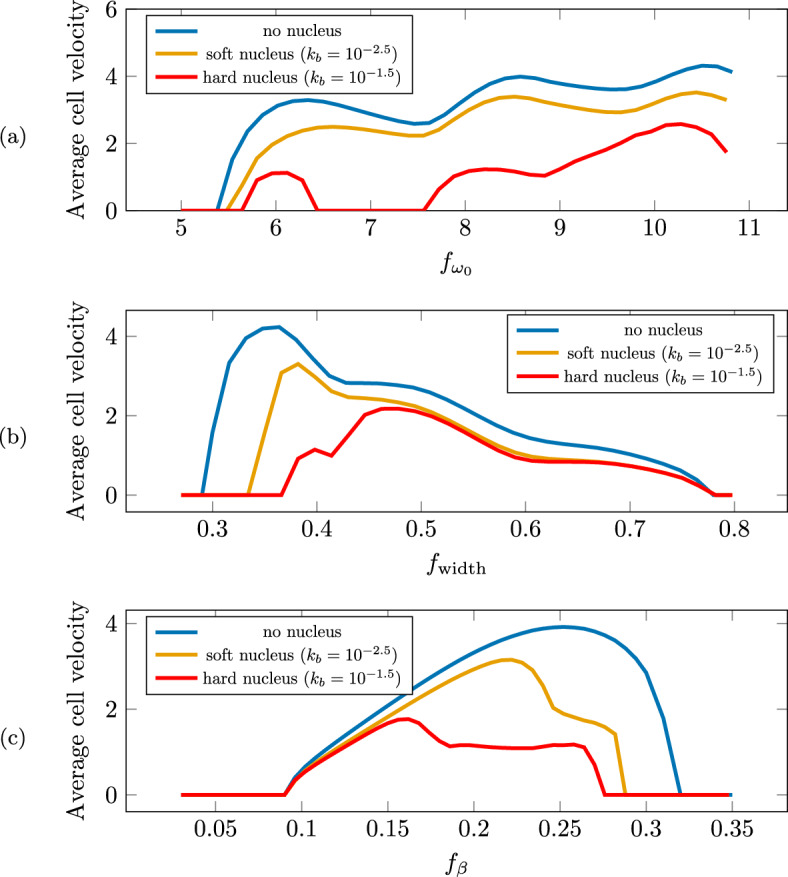
(Color figure online) Comparison of the motilities associated with cell with and without nucleus, for three different channel parameters. Top: Wave number of the channel pattern $f_{\omega _0}$. Middle: mean width of the channel $f_\text {width}$. Bottom: depth of the pattern $f_\beta$. Model parameters are summed up in Table 1

$^\textrm{a}$Inverse of the width of the approximating potential, see Fig. 3

$$^\textrm{b}$$Figure 6

$$^\textrm{c}$$Figure 5

$$^\textrm{d}$$Figure 7

Finally, in Fig. 7, we consider the cell average velocity for varying values of the parameters describing the channel geometry and we compare the results obtained either with or without the description of the nucleus. In particular, we first consider the pulsation of the channel in determining the cell capability to move. As observed for the cell without the nucleus (blue line in Fig. 7a) the cell is able to migrate only if the channel is sufficiently structured, i.e. for channel pulsation above a minimum threshold, $f_{\omega _\textrm{min}}$.

The value of the threshold $f_{\omega _\textrm{min}}$ is only slightly influenced by the presence of the nucleus. The latter mainly affects the cell average speed, which decreases in the presence of the nucleus. Specifically, the cell speed decreases when increasing the value of $k_b$ (see the yellow and red curves). However, for high values of $k_b$, the cell gets stuck (see the red curve in Fig. 7a) even for intermediate values of channel pulsation, since the hourglass nuclear deformation is impeded by the high bending modulus and the space between two subsequent constriction is enough to host the cell nucleus. Increasing the channel pulsation further, the nucleus characterised by a high bending modulus is again able to migrate since the space between two constriction becomes restrictive for the preservation of the nuclear area and the nucleus can deform acquiring an ellipsoidal shape, without following the structure of the channel walls. We remark that, since the discretisation of the cell membrane is finite, the channel structure will not be well resolved as $f_ {\omega _0}$ is taken larger and larger. This explains why the first plot in Fig. 7a is cut on the right hand side. Note that this will occur for any discretisation size.

The influence of the channel width on the cell velocity is analysed in Fig. 7b: without considering the nucleus (blue curve), the cell can move even for smaller channel width and this “physical limit of cell migration” is related to the capability of the cell envelope and the cytosol to deform and enter even small gaps. Indeed, the size of the smallest constriction inside the channel is $2(f_\textrm{width} - f_{\beta })$. When we include the nucleus, the cell is no longer able to move inside channels with a neck which is too narrow (i.e. small values of $f_\textrm{width}$), since the nucleus hinders cell capability to deform (yellow curve). Raising the bending modulus (red curve), the cell can move inside the channel for large values of $f_\textrm{width}$ only. On the other hand, the upper limit is the same in all three cases, since it is related to the capability of the whole cell to maintain the contact with the channel wall and it is not affected by the presence and the mechanical properties of the nucleus. Furthermore, the plots in Fig. 7b show the well known bimodal behaviour of cell velocity for varying channel width (Ulrich et al. 2009; DiMilla et al. 1993; Kuntz and Saltzman 1997): cells cannot migrate both inside very small channels (since deformations would be too large) and inside very large channels (since the cell does not touch the boundary of the channel. The velocity of cell motion is also in this case slowed down by the presence of the nucleus. Specifically, the speed decreases for increasing values of $k_b$, as previously observed.

The bimodal behaviour in the cell velocity can be observed also when changing the value of $f_{\beta }$ and keeping the channel width fixed (see Fig. 7c). In this case, the upper limit of $f_{\beta }$ is related to size of the small constriction that the cell could enter in order to move inside the channel and thus it is highly influenced by the presence of the nucleus and by its mechanical properties. On the other hand, when $f_\beta$ is small the channel is almost flat and the cell cannot migrate, independently on the presence of the nucleus and its mechanical properties. Therefore, the lower limit for $f_{\beta }$ is the same both for the cell with and without the nucleus, since it is related to the mechanism of motion, which requires a sufficient structure of the lateral walls.

## Conclusions

In this work we have proposed a continuous mechanical model describing single cell adhesion-independent migration inside restrictive 3D environments. The cell migration is driven by a local imbalance in the polymerization and depolymerization of the actin network underneath the cell membrane, which induces a cortex flow. The cell shape is determined by the balance of cytoplasmic pressure, elastic behaviour of the cell cortex and interactions with the subcellular elements and the channel walls. Differently from previous works (Jankowiak et al. 2020), the influence of the nucleus in the process of cell migration is explicitly taken into account by including an inner nuclear membrane, connected to the cell membrane by the microtubule structure, responsible for the positioning of the nucleus inside the cell. The nucleus shows a resistance to bending, to stretching and to changes in the area enclosed by the nuclear membrane and it becomes the limiting factor in determining the ability of the cell to migrate across small neckings of the channel. Therefore, the proposed model represents an advancement with respect to the state of the art, since it provides a purely mechanical description of adhesion-free migration, without requiring external chemical stimuli as done in Lee et al. (2017), Chen et al. (2018) and Cao et al. (2016). It also allows testing the influence of nucleus mechanical properties in the determination of the physical limit of cell migration, which has been neglected in previous models (Wu et al. 2018; Stotsky and Othmer 2022; Kaoui et al. 2008; Moure and Gomez 2017, 2018; Jankowiak et al. 2020).

The model equations have been discretised and solved numerically in order to simulate the process in a 2D geometry, corresponding to a section of the 3D channel with lateral structured walls and top and bottom flat walls. To formulate an efficient and stable (under appropriate time step restrictions) discretisation scheme, we adapt the approach proposed in Mikula and Ševčovič (2004) and Beneš et al. (2009) to our model.

The numerical simulations reproduce qualitatively the behaviour observed in the biological experiments (Reversat et al. 2020), where it is pointed out that adhesion-independent migration needs both confinement and sufficiently structured channel walls. This behaviour is purely related to the cell membrane behaviour and it is not influenced by the presence of the nucleus, since the threshold for the channel structure depth (i.e. lowest value of $f_\beta$ that allows cell motion) predicted by our model is the same of the one predicted by the model developed in Jankowiak et al. (2020). However, the cell speed inside the channel and the physical limit of cell migration (i.e. the size of the smallest opening in the channel) substantially depends on the presence of the nucleus and on its mechanical properties, as demonstrated by the parametric study showing the dependency on geometric properties of the channel and on the mechanical properties of the nucleus. In particular, in agreement with biological experiments (Wolf et al. 2003, 2013), we observe that a little-deformable nucleus is a limiting factor for cell migration inside restrictive environments. Indeed, by keeping both the geometry of the channel and the cell membrane parameters fixed, we can show the transition from a migrating cell to a non-moving cell by changing the mechanical properties of the nucleus only (i.e. increasing the bending modulus $k_b$ and the elastic area constraint $\mu _n$).

Furthermore, the sensitivity analysis of the mechanical parameters of the nucleus predicts the optimal nuclear deformability, for which the cell reaches its maximal speed, for low values of $k_b$ and intermediate values of $\mu _n$.

Without claiming to provide quantitative numerical measurements, the results presented in this work are a proof of concept for a comprehensive model of single cell adhesion-independent migration taking into account cell and nucleus mechanics. Nonetheless, these results need to be validated quantitatively by comparing the predicted evolution with the actual spatio-temporal evolution of the moving cell inside a structured channel, by also keeping track of nuclear deformations. Therefore, additional work is needed to investigate more realistic in vitro and in vivo conditions, in order to quantitatively validate the model.

From a modelling point of view, this study has to be seen as a first step and several components would benefit from a more detailed treatment. In particular, the role of the compensating force, that can be attributed to the transport of actin monomers and their polymerization, certainly requires further studies. For example, one could include an explicit description of the acto-myosin machinery and its influence in cell polymerization. Another interesting direction would be to take into account the resistance to deformation of the cortex, which can then be considered as visco-elastic and not only elastic. Moreover, the mechanical description of the nucleus is kept rather simple in the present model and a detailed description of both the deformations occurring inside the nucleus and the exchange of liquid between the nucleus and the surrounding cytosol during the whole process of cell migration is still missing. Finally, the microtubule description is kept really simple and it does not take into account some important biological observations, such as the bending of some MTs close to the cell cortex, the dynamic instability process, the detailed description of the anchorage of the MT structure to the cell cortex and to the intermediate filaments network surrounding the nucleus membrane. The inclusion of these biological observation inside the model is fundamental to give a biologically sound description of the active and passive force exerted by the MTs and of their role in positioning the cell nucleus, while maintaining the cell shape. From the biological point of view, it would be interesting to perform ad-hoc experiments to quantitatively verify the model prediction. Indeed, even though most of the model parameters could be in principle measured or at least estimated from numerical experiments, it is not possible to derive all of them from the biological observations reported in the literature. Therefore, future works will certainly be addressed to the fitting of the model parameters with experimental data.

Taking all these effects into account could lead to a more comprehensive understanding of the multiple factors involved in determining the physical limit of cell migration during non-proteolytic migration of cells.

### Supplementary Information

Below is the link to the electronic supplementary material.

Supplementary file 1 (mp4 317 KB)

Supplementary file 2 (mp4 391 KB)

Supplementary file 3 (mp4 314 KB)

Supplementary file 4 (mp4 395 KB)

Supplementary file 5 (mp4 121 KB)

## Appendix A: Nuclear Energy Variation

We here compute the variation $\delta E_{n}$ of the nuclear membrane energy function, in order to obtain the force acting on the nuclear membrane. We named

$$\begin{aligned} \begin{aligned}&E_{n}^{(1)} = \dfrac{k_b}{2} \int _{\Gamma _n} K^2 \,\textrm{d}\ell \, \quad E_{n}^{(2)} =\lambda \int _{\Gamma _n} \, \textrm{d}\ell \,; \quad E_{{n}}^{(3)} =\Delta p_n {\int _{\Omega _n}} \,\textrm{d}A \,; \\&E_{{n}}^{(4)} =\mu _n \left( {\int _{\Omega _n}}\,\textrm{d}A -A_{n}^* \right) ^2 \end{aligned} \end{aligned}$$

so that $E_{{n}}= E_{{n}}^{(1)} + E_{{n}}^{(2)} + E_{{n}}^{(3)} + E_{{n}}^{(4)}$ and $\delta E_{{n}}= \delta E_{{n}}^{(1)} + \delta E_{{n}}^{(2)} + \delta E_{{n}}^{(3)} +\delta E_{{n}}^{(4)}$. In order to compute $\delta E_{{n}}^{(j)}$, with $j=1,2,3,4$, it is convenient to use the parametrization of the curve with respect to $\sigma$, since $\textrm{d}\ell$ will undergo variations as well for displacement of *Y*.

The first energy term with respect to $\sigma$ (assuming $K_0=0$) reads

$$\begin{aligned} E_{{n}}^{(1)} = \dfrac{k_b}{2} {\int _{\Gamma _n}}K^2 \,\textrm{d}\ell = \dfrac{k_b}{2} {\int _{{\mathbb {T}}_1}} \left( (\partial _{\sigma \sigma } Y)^2 - \left( \dfrac{\partial _{\sigma \sigma } Y \cdot \partial _{\sigma } Y}{\vert \partial _{\sigma } Y\vert }\right) ^2\right) \vert \partial _{\sigma } Y\vert ^{-3}\,\textrm{d}\sigma \end{aligned}$$

and its variation is (Kaoui et al. 2008)

$$\begin{aligned} \dfrac{\delta E_{{n}}^{(1)}}{\delta Y} = - k_b \left| \partial _{\sigma } Y \right| \left( \dfrac{\partial ^2 K}{\partial \ell ^2} + \dfrac{1}{2} K^3 \right) N \, . \end{aligned}$$

The variation of the second energy term can be easily computed

$$\begin{aligned} \delta E_{{n}}^{(2)}&= \lambda {\int _{\Gamma _n}}\delta (\textrm{d}\ell )= \lambda {\int _{{\mathbb {T}}_1}} \delta (\left| \partial _{\sigma } Y \right| ) \, \textrm{d}\sigma = \lambda {\int _{{\mathbb {T}}_1}} \dfrac{\partial _{\sigma } Y }{\left| \partial _{\sigma } Y \right| } \cdot \delta (\partial _{\sigma } Y ) \, \textrm{d}\sigma \\&=\quad -\lambda {{\int _{{\mathbb {T}}_1}}}\partial _{\sigma } \left( \dfrac{\partial _{\sigma } Y }{\left| \partial _{\sigma } Y \right| } \right) \cdot \delta Y \, \textrm{d}\sigma \\&= -\lambda {{\int _{{\mathbb {T}}_1}}}\partial _{\sigma } T \cdot \delta Y \, \textrm{d}\sigma = \lambda {\int _{\Gamma _n}}K \, N \cdot \delta Y \left| \partial _{\sigma } Y \right| \, \textrm{d}\sigma \, , \end{aligned}$$

and thus

$$\begin{aligned} \dfrac{\delta E_{{n}}^{(2)}}{\delta Y}&= \lambda \left| \partial _{\sigma } Y \right| K \, N . \end{aligned}$$

In order to calculate $\delta E_{{n}}^{(3)}$ and $\delta E_{{n}}^{(4)}$, we use the identity

$$\begin{aligned} E_{{n}}^{(3)}&= {\int _{\Omega _n}} \, \textrm{d}A= \dfrac{1}{2} {\int _{\Gamma _n}}Y \cdot N \, \textrm{d}\ell =-\dfrac{1}{2} {\int _{\Gamma _n}}Y \cdot \partial _{\ell } Y ^\perp \, \textrm{d}\ell =- \dfrac{1}{2} {{\int _{{\mathbb {T}}_1}}}Y \cdot \partial _{\sigma } Y ^\perp \, \textrm{d}\sigma \end{aligned}$$

so that

$$\begin{aligned} \delta E_{{n}}^{(3)}&= - \Delta p_n {{\int _{{\mathbb {T}}_1}}}\partial _{\sigma } Y^\perp \cdot \delta Y \textrm{d}\sigma \, ,\\ \delta E_{{n}}^{(4)}&= - \mu _n \left( {{\int _{{\mathbb {T}}_1}}}Y \cdot \partial _{\sigma } Y ^\perp \, \textrm{d}\sigma -A_{n}^* \right) {{\int _{{\mathbb {T}}_1}}}\partial _{\sigma } Y^\perp \cdot \delta Y \textrm{d}\sigma \end{aligned}$$

Therefore, we have

$$\begin{aligned} \dfrac{\delta E_{{n}}^{(3)}}{\delta Y}&= - \Delta p_n \partial _{\sigma } Y^\perp = \Delta p_n \left| \partial _{\sigma } Y \right| N \, ,\\ \dfrac{\delta E_{{n}}^{(4)}}{\delta Y}&= - \mu _n \left( {{\int _{{\mathbb {T}}_1}}}Y \cdot \partial _{\sigma } Y ^\perp \, \textrm{d}\sigma -A_{n}^* \right) \partial _{\sigma } Y^\perp \\&= \mu _n \left( {{\int _{{\mathbb {T}}_1}}}Y \cdot \partial _{\sigma } Y ^\perp \, \textrm{d}\sigma -A_{n}^* \right) \left| \partial _{\sigma } Y \right| N \end{aligned}$$

Finally, the nuclear membrane mechanical force reads

$$\begin{aligned} F_n= & {} k_b \left( \dfrac{\partial ^2 K}{\partial \ell ^2} + \dfrac{1}{2} K^3 \right) N - \lambda K \, N - \Delta p_n N \\{} & {} \quad - \mu _n \left( {{\int _{{\mathbb {T}}_1}}}Y \cdot \partial _{\sigma } Y ^\perp \, \textrm{d}\sigma -A_{n}^* \right) N \end{aligned}$$

## Appendix B: Discretisation and Quadrature Formulae for the Centrosome and the Microtubule Structure

Assume that *X* is a polygon with nodes $X_i = X(\theta _i)$ such that

$$\begin{aligned} X(\theta _i) = X_c + r_i \begin{pmatrix} \cos \theta _i \\ \sin \theta _i \end{pmatrix}, \text { so that } \vert X(\theta _i) - X_c\vert = r_i, \end{aligned}$$

where $X_c$ is the centrosome. Then the line segment between $X_i$ and $X_{i+1}$ has the following equation in polar coordinates

$$\begin{aligned} r_i(\theta )(a_i \cos \theta + b_i \sin \theta ) = 1 \end{aligned}$$

where

$$\begin{aligned} a_i = \frac{r_{i+1} \sin \theta _{i+1} - r_{i} \sin \theta _{i}}{r_{i} r_{i+1} \sin (\theta _{i+1} - \theta _{i})}, \text { and } b_i = -\frac{r_{i+1} \cos \theta _{i+1} - r_{i} \cos \theta _{i}}{r_{i} r_{i+1} \sin (\theta _{i+1} - \theta _{i})}, \end{aligned}$$

Then we have the following identities:

$$\begin{aligned} \int _0^{2\pi } \vert X_c - X_\theta \vert ^2 \textrm{d}\theta&= \sum _i \int _{\theta _i}^{\theta _{i+1}} \frac{\textrm{d}\theta }{(a_i \cos \theta + b_i \sin \theta )^2} \\&= \sum _i (a_i^2 \cot \theta + a_i b_i)^{-1} \Big \vert _{\theta _i}^{\theta _{i+1}} \\ \int _0^{2\pi } \vert X_c - X_\theta \vert \textrm{e}_\theta ^\perp \textrm{d}\theta&= \sum _i \int _{\theta _i}^{\theta _{i+1}} \begin{pmatrix}-\sin \theta \\ \cos \theta \end{pmatrix} \frac{\textrm{d}\theta }{(a_i \cos \theta + b_i \sin \theta )} \\&= \sum _i (a_i^2+b_i^2)^{-1} \begin{pmatrix} - a_i \log (r_i(\theta )) - b_i \theta \\ - b_i \log (r_i(\theta )) + a_i \theta \end{pmatrix}\Bigg \vert _{\theta _i}^{\theta _{i+1}} \end{aligned}$$

In order to integrate function over the cortex $f(\Pi _{MT}(\theta )) = f(s_{MT}(\theta ))$ w.r.t. $\theta$, we need to know $\Pi _{MT}(\theta )$ and ${\Pi _{MT}^{-1}}'(s)$ in order to perform the change of variables

34 $$\begin{aligned} \int _{\theta _i}^{\theta _{i+1}} f(\Pi _{MT}(\theta )) \textrm{d}\theta = \int _{s_i}^{s_{i+1}} f(s) \vert {\Pi _{MT}^{-1}}'(s)\vert \textrm{d}s \end{aligned}$$

The expression of $\vert {\Pi _{MT}^{-1}}'(s)\vert$ is derived from (7):

$$\begin{aligned} \vert {\Pi _{MT}^{-1}}'(s)\vert&= \frac{\vert n(s)\cdot (X(s) - X_c)\vert }{\vert X(s)-X_c\vert ^2} \\&= \left| (X_{i+1} - X_i)^\perp \cdot \frac{X_i - X_c + \lambda (X_{i+1} - X_i)}{\vert X_i - X_c + \lambda (X_{i+1} - X_i)\vert ^2}\right| \\&= \frac{\vert (X_{i+1} - X_i)^\perp \cdot (X_i - X_c)\vert }{\vert X_i - X_c + \lambda (X_{i+1} - X_i)\vert ^2} \end{aligned}$$

If in (34) we take a piecewise linear approximation for *f*, we can compute the corresponding integral if we can compute

$$\begin{aligned} \int _0^1 \frac{\lambda ^\alpha \textrm{d}\lambda }{\vert X_i-X_c + \lambda (X_{i+1}-X_i)\vert ^2}, \quad \alpha \in \left\{ 0,1\right\} , \end{aligned}$$

which can be put in the form

$$\begin{aligned} \int _0^1 \frac{\lambda ^\alpha \textrm{d}\lambda }{c_0 + c_1 \lambda + c_2 \lambda ^2} \end{aligned}$$

where

$$\begin{aligned}&c_0 = \vert X_i - X_c\vert ^2\,,\quad c_1 = 2(X_{i+1}-X_i)\cdot (X_i - X_c)\,,\quad c_2 = \vert X_{i+1} - X_i\vert ^2\\&\alpha = 0: \frac{2\tan ^{-1}\frac{2c_2\lambda + c_1}{\sqrt{4c_2c_0-c_1^2}}}{\sqrt{4c_2c_0-c_1^2}} \\&\alpha = 1: \frac{1}{2c_2}\left[ \log \left( \lambda (c_2\lambda +c_1)+c_0\right) -\frac{2c_1 \tan ^{-1}\frac{2c_2\lambda +c_1}{\sqrt{4c_2c_0-c_1^2}}}{\sqrt{4c_2c_0-c_1^2}}\right] \\&\alpha = 2: \frac{1}{2c_2^2} \left[ 2c_2\lambda - c_1\log \left( \lambda (c_2\lambda +c_1)+c_0)\right) + \tfrac{2\left( c_1^2-2c_2c_0\right) \tan ^{-1}\frac{2c_2\lambda +c_1}{\sqrt{4c_2c_0-c_1^2}}}{\sqrt{4c_2c_0-c_1^2}} \right] \end{aligned}$$

The last expression we need to compute is of the kind $\int \vert X_\theta - X_c\vert \textrm{e}_\theta ^\perp f(s_{MT}(\theta )) \textrm{d}\theta$. To do so, we notice that

$$\begin{aligned} \vert \Pi _{MT}^{-1}(s)\vert \vert X_c - X_\theta \vert \textrm{e}_\theta ^\perp = \vert (X_{i+1} - X_i)^\perp \cdot (X_i - X_c)\vert \frac{(X_i - X_c + \lambda \left( X_{i+1} - X_i\right) )^\perp }{\vert X_i - X_c + \lambda (X_{i+1} - X_i)\vert ^2}, \end{aligned}$$

and the corresponding integral can be computed directly, using the case $\alpha = 2$ above.

## Appendix C: The Nuclear Membrane Evolution Equations

### The Formulation of Mikula–Ševčovič

The nuclear membrane $\Gamma _n(t)$ can be either parameterized by the arc length $\ell$ or by $\sigma$ which always span the same interval [0, 1]

$$\begin{aligned} \Gamma _n(t) {:}{=} \left\{ Y(t, \ell ): \ell \in [0, L_n(t)] \right\} = \left\{ Y(t, \sigma ): \sigma \in [0, 1] \right\} \,. \end{aligned}$$

Therefore, we can define the local length element $g {:}{=} \vert \partial _\sigma Y(\sigma )\vert$, i.e. $\textrm{d}\ell = g \textrm{d}\sigma$ The curvature is denoted by *K* and $\nu$ is the tangential angle, so that $T = (\cos \nu , \sin \nu )$ and $N = (\sin \nu , -\cos \nu )$. The region enclosed by $\Gamma _n$ is called $\Omega _n$ and $\textrm{d}A$ denotes its surface measure.

The curve’s total energy $E_n$ is given by:

$$\begin{aligned} E_n^{TOT} = E_{{n}}^{(1)}+ E_{{n}}^{(2)}+ E_{{n}}^{(3)} + \int _{\Gamma _n} W(Y(\ell )) \textrm{d}\ell , \end{aligned}$$

where the first three terms are given by (A1), while *W* denotes a potential, which combines the contribution related to the nuclear membrane surface tension (i.e. $E_{{n}}^{(4)}$ given by (A1)), the contact interaction with the cell cortex and the elastic constraint linking the nucleus with the centrosome. Following the method proposed by Mikula and Ševčovič (2004), the evolution of the planar curve $\Gamma _n$ is, then, given by:

35 $$\begin{aligned} {\left\{ \begin{array}{ll} \partial _t K &{}= -\partial _\ell ^2 \beta + \alpha \partial _\ell K - K^2 \beta \\ \partial _t \nu &{}= -\partial _\ell \beta + \alpha K \\ \partial _t g &{}= g K \beta + g\partial _\ell \alpha \\ \partial _t Y &{}= \alpha T + \beta N, \end{array}\right. } \end{aligned}$$

where *K*, $\nu$ and $g = \vert \partial _\sigma Y\vert$ are the curvature, tangential angle and local length element, respectively. Keeping in mind the previous governing equations, it is possible to calculate the time derivative of the energy functional *E*, formally given by

$$\begin{aligned} \begin{aligned} \frac{d}{dt} E_n^{TOT}&= -k_b \int _{\Gamma _n} (\partial _\ell ^2 K + \frac{1}{2}K^3) \beta + \Delta p_n \int _{\Gamma _n} \beta + \mu _n \int _{\Gamma _n} (A_n -A_{n}^*) \beta \\&\quad + \int _{\Gamma _n} \nabla W \cdot (\alpha T + \beta N) + \int _{\Gamma _n} (W K \beta - \alpha \partial _\ell W ) \\&= -k_b \int _{\Gamma _n} (\partial _\ell ^2 K + \frac{1}{2}K^3) \beta \\&\quad + \Delta p_n \int _{\Gamma _n} \beta + \int _{\Gamma _n} (\nabla W \cdot N)\beta + \int _{\Gamma _n} W K \beta . \end{aligned} \end{aligned}$$

Then, the choice of $\beta$ is made to maximise the decrease of energy due to the normal component, whereas the tangential velocity $\alpha$, in principle, is a free parameter. In this paper, we refer to the asymptotically uniform tangential redistribution derived in Mikula and Ševčovič (2004) and Beneš et al. (2009), to impose the evolution of $\alpha$ in order to avoid nodes concentrating on points, which would lead to poor approximation and eventually inversion of ill-conditioned matrices. Therefore, we take

36 $$\begin{aligned} \partial _\ell \alpha&= -K\beta + \langle K \beta \rangle + (L_n/g-1) \zeta \end{aligned}$$

37 $$\begin{aligned} \beta&= k_b \left( \partial _\ell ^2 K + \frac{1}{2} K^3\right) - \Delta p_n - \mu _n (A_n -A_{n}^*) - \nabla W \cdot N - W K \,, \end{aligned}$$

where $\langle \;. \; \rangle = L_n^{-1} \int _{\Gamma _n}. \; \textrm{d}\ell$ denotes the average over the whole curve $\Gamma _n$, which makes (36) a non-local equation, whereas $\zeta >0$ is a given positive constant.

Recalling the identity:

$$\begin{aligned} \partial _\ell ^4 Y&= -(\partial _\ell ^2 K) N - 2 \partial _\ell K \partial _\ell N - K \partial _\ell ^2 N = -(\partial _\ell ^2 K) N - 2 K \partial _\ell K T - K \partial _\ell (K T) \\&= -(\partial _\ell ^2 K) N - 3 K \partial _\ell K T - K^2 \partial _\ell T = -(\partial _\ell ^2 K) N - \frac{3}{2}\partial _\ell K^2 \partial _\ell Y - K^2 \partial _\ell ^2 Y \end{aligned}$$

and $\partial _\ell ^2 Y = -K N$, we have

$$\begin{aligned} \left( \partial _\ell ^2 K + \frac{1}{2}K^3\right) N&= -\partial _\ell ^4 Y - \frac{3}{2} K^2 \partial _\ell ^2 Y - \frac{3}{2} \partial _\ell K^2 \partial _\ell Y \\&= -\partial _\ell ^4 Y - \frac{3}{2} \partial _\ell \left( K^2 \partial _\ell Y\right) \, , \end{aligned}$$

so that, setting $g = \exp \eta$, we can rewrite (35) as

38 $$\begin{aligned} {\left\{ \begin{array}{ll} \partial _t K &{}= - k_b \left( \partial _\ell ^4 K + \frac{1}{2} \partial _\ell ^2(K^3)\right) + \partial _\ell (\alpha K) - K(K\beta + \partial _\ell \alpha ) \\ &{}\quad + \partial _\ell ^2 (\nabla W \cdot N) + \partial _\ell ^2 (W K) \\ \partial _t \nu &{}= -k_b \partial _\ell ^4 \nu - \frac{k_b}{2}\partial _\ell (\partial _\ell \nu )^3 + \partial _\ell (\nabla W\cdot N) + \partial _\ell (W \partial _\ell \nu ) + \alpha \partial _\ell \nu \ \\ \partial _t \eta &{}= K \beta + \partial _\ell \alpha \\ \partial _t Y &{}= \alpha \partial _\ell Y - k_b \left( \partial _\ell ^4 Y + \frac{3}{2} \partial _\ell (K^2 \partial _\ell Y)\right) + W \partial _\ell ^2 Y - \Delta p_n \, N \\ &{}\quad - \mu _n (A_n -A_{n}^*) N - \left( \nabla W \cdot N \right) N \end{array}\right. } \end{aligned}$$

We remark that the second equation in (38) has been obtained remembering that $K = \partial _\ell \nu$.

### Quadrature Formulae for the Mikula Formulation

We can now write the corresponding discretised equations of (38). To do so, we discretise the fixed parameterization interval [0, 1] uniformly in $N_2$ subintervals, each of equal length $h=1/N_2$ and indexed by $i \in \{0, N_2-1\}$. Time is also discretised with a time-step $\Delta t$. The discrete time is indexed by $j\in {\mathbb {N}}$, so that $t_j = j\, \Delta t$. In what follows, indices corresponding to space (resp. time) will be in subscript (resp. superscript), so that the point $Y(ih, j\Delta t)$ is written $Y_i^j$. The measure of the finite element $[Y_{i-1}^j, Y_i^j]$ at time $t_j$, is given by $r_i = \vert Y_{i-1} - Y_i\vert$. We also introduce the dual finite element $[{\tilde{Y}}_{i}^j, {\tilde{Y}}_{i+1}^j]$, containing the node $Y_{i}^j$, where ${\tilde{Y}}_{i}^j= \frac{Y_{i-1}^j+Y_i^j}{2}$. The length of the dual finite element is denoted by $q_i^j = \frac{1}{2} \left( r_i^j + r_{i+1}^j\right)$. Then, the system of equations (36)–(37)–(38) is solved for the discrete quantities $\alpha _i^j$, $\beta _i^j$, $K_i^j$, $\nu _i^j$, $\eta _i^j$, $Y_i^j$. In particular $\alpha _i^j$ denotes the tangential velocity of the node $Y_i^j$, whereas $\beta _i^j$, $K_i^j$, $\nu _i^j$, $\eta _i^j$ are piecewise constant approximations of the corresponding quantities on the finite element $[Y_{i-1}^j, Y_i^j]$. The dependent variables *W*(*Y*) and $\nabla W(Y)$ are consequently evaluated numerically at nodes, i.e. $W_i^j=W(Y_i^j)=W(Y(ih, j\Delta t))$, whereas the tangential and normal vectors are approximated on the finite element $[Y_{i-1}^j, Y_i^j]$, by the quantities $T_i^j = (\cos \nu _i^j, \sin \nu _i^j)^T$ and $N_i^j = (\sin \nu _i^j, - \cos \nu _i^j)^T$.

We first integrate (36) on the finite element $[Y_{i-1}, Y_i]$

$$\begin{aligned} \int _{Y_{i-1}}^{Y_i} \partial _\ell \alpha \textrm{d}\ell = \alpha _{i} - \alpha _{i-1}&= \int _{Y_{i-1}}^{Y_i} -K \beta + \langle K \beta \rangle + \left( L/g - 1\right) \omega \, \textrm{d}\ell \\&\simeq r_i \left( -K_i \beta _i + \langle K \beta \rangle \right) + \left( L/N_2 - r_i\right) \omega \,. \end{aligned}$$

and we discretise in time to get

$$\begin{aligned} \alpha _i^j = \alpha _{i-1}^j + r_i^{j-1} \left( -K_i^{j-1} \beta _i^{j-1} + B^{j-1}\right) + \left( L^{j-1}/ N_2 - r_i^{j-1}\right) \omega , \end{aligned}$$

where $L^j = \sum _l r_l^j\,, \quad B^j = (L^j)^{-1} \sum _l r_l^j K_l^j \beta _l^j$ and $\beta _i^j$ obtained by the discretization of (37), i.e.

$$\begin{aligned} \beta _i^j= & {} \frac{k_b}{r_i^j} \left( \frac{K_{i+1}^j - K_i^j}{q_i^j} - \frac{K_i^j - K_{i-1}^j}{q_{i-1}^j}\right) + \frac{1}{2} k_b (K_i^j)^3 - \Delta p_n \\{} & {} - N_i^{j}\cdot \nabla {\tilde{W}}_i^{j} - {\tilde{W}}_i^{j} K_i^{j}. \end{aligned}$$

This determines $\alpha _i^j$ for all *j*, provided a closure for $\alpha _0^j$. It is possible to assume $\alpha _0^j=0$, i.e. the point $Y_0^j$ moves along the normal (Beneš et al. 2009). The local length of the finite element is updated in a similar way, using (38)$_{c}$:

$$\begin{aligned} r_i^{j-1} \frac{\eta _i^j - \eta _i^{j-1}}{\Delta t} = r_i^{j-1} K_i^{j-1} \beta _i^{j-1} + \alpha _i^j - \alpha _{i-1}^j, \end{aligned}$$

after which we set $r_i^j = \exp (\eta _i^j)$. Integrating Eq. (38)$_{a}$ on $[Y_{i-1}, Y_i]$ we get

39 $$\begin{aligned} \begin{aligned} \int _{Y_{i-1}}^{Y_i} \partial _t K&= \int _{Y_{i-1}}^{Y_i} \Big [-k_b \partial _\ell ^4 K - \frac{k_b}{2} \partial _\ell ^2 (K^3) + \partial _\ell (\alpha K) - K(K\beta + \partial _\ell \alpha ) \\&\qquad \qquad + \partial _\ell ^2 (\nabla W\cdot N) + \partial _\ell ^2 (W K) \Big ] \textrm{d}\ell \\ r_i^j \partial _t K_i&= -k_b [\partial _\ell ^3 K]_{Y_{i-1}}^{Y_i} -\frac{k_b}{2} [\partial _\ell (K^3)]_{Y_{i-1}}^{Y_i} + [\alpha K]_{Y_{i-1}}^{Y_i} \\&\quad - K_i(K_i r_i \beta _i + (\alpha _i - \alpha _{i-1})) \\&\quad + [\partial _\ell ( \nabla W \cdot N)]_{Y_{i-1}}^{Y_i} + [\partial _\ell (W K)]_{Y_{i-1}}^{Y_i} \\&= -k_b [\partial _\ell ^3 K]_{Y_{i-1}}^{Y_i} -\frac{k_b}{2} [\partial _\ell (K^3)]_{Y_{i-1}}^{Y_i} + [\alpha K]_{Y_{i-1}}^{Y_i} \\&\quad - K_i(K_i r_i \beta _i + (\alpha _i - \alpha _{i-1})) \\&\quad + [\partial _\ell ( \nabla W \cdot N)]_{Y_{i-1}}^{Y_i} + [\partial _\ell (W K)]_{Y_{i-1}}^{Y_i} \end{aligned} \end{aligned}$$

If we approximate the time derivative as $\Delta t^{-1} (K_i^j - K_i^{j-1})$, $K_i^j$ solves a linear system of the form

40 $$\begin{aligned} a_i^j K_{i-2}^j + b_i^j K_{i-1}^j + c_i^j K_i^j + d_i^j K_{i+1}^j + e_i^j K_{i+2}^j = f_i^j. \end{aligned}$$

Then, we have to solve the tangent angle equation (38)$_{b}$, using the following approximation

41 $$\begin{aligned} \int _{Y_{i-1}}^{Y_i} \partial _t \nu&= \int _{Y_{i-1}}^{Y_i} \Big [- k_b \partial _\ell ^4 \nu - \frac{k_b}{2} \partial _\ell (\partial _\ell \nu )^3 + \partial _\ell (\nabla W \cdot N) \nonumber \\&\qquad \qquad + \partial _\ell (W \partial _\ell \nu ) + \alpha \partial _\ell \nu \Big ] \textrm{d}\ell \nonumber \\ r_i^j \partial _t \nu _i&= -k_b [\partial _\ell ^3 \nu ]_{Y_{i-1}}^{Y_i} -\frac{k_b}{2} [(\partial _\ell \nu )^3]_{Y_{i-1}}^{Y_i} + [\nabla W \cdot N ]_{Y_{i-1}}^{Y_i} + [\alpha \nu ]_{Y_{i-1}}^{Y_i} \nonumber \\&\quad + [W \partial _\ell \nu ]_{Y_{i-1}}^{Y_i} - \nu _i (\alpha _i - \alpha _{i-1}) \end{aligned}$$

and approximating the time differential by finite differences, we obtain the following system for $\nu _i^j$

42 $$\begin{aligned} {\mathcal {A}}_i^j \nu _{i-2}^j + {\mathcal {B}}_i^j \nu _{i-1}^j + {\mathcal {C}}_i^j \nu _i^j + {\mathcal {D}}_i^j \nu _{i+1}^j + {\mathcal {E}}_i^j \nu _{i+2}^j = {\mathcal {F}}_i^j. \end{aligned}$$

Finally, the discretisation of the equation for *Y* (38)$_{d}$ is obtained by integrating on the dual element $[{\tilde{Y}}_{i+1}, {\tilde{Y}}_i]$, i.e.

$$\begin{aligned} \int _{{\tilde{Y}}_{i}}^{{\tilde{Y}}_{i+1}} \partial _t Y \textrm{d}\ell&= \int _{{\tilde{Y}}_{i}}^{{\tilde{Y}}_{i+1}} \Bigg [\alpha \partial _\ell Y - k_b \left( \partial _\ell ^4 Y + \frac{3}{2} \partial _\ell (K^2 \partial _\ell Y)\right) \\&\qquad + W \partial _\ell ^2 Y - \Delta p_n \, N - \mu _n (A_n -A_{n}^*) N - \left( \nabla W \cdot N \right) N \Bigg ] \textrm{d}\ell \end{aligned}$$

So that the complete semi-discrete equation is:

43 $$\begin{aligned} q_{i} \partial _t Y_i= & {} \alpha _i ({\tilde{Y}}_{i+1}-{\tilde{Y}}_{i}) -k_b [\partial _\ell ^3 Y]_{{\tilde{Y}}_{i}}^{{\tilde{Y}}_{i+1}} - \frac{3}{2} k_b [K^2 \partial _\ell Y]_{{\tilde{Y}}_{i}}^{{\tilde{Y}}_{i+1}}+ W_ i[ \partial _\ell Y]_{{\tilde{Y}}_i}^{{\tilde{Y}}_{i+1}} \nonumber \\{} & {} \quad - \frac{\Delta p_n + \mu _n (A_n -A_{n}^*) }{2} \left( r_{i+1} N_{i+1} + r_{i} N_{i} \right) \nonumber \\{} & {} \quad - \frac{1}{2}(r_{i+1}(N_{i+1} \cdot \nabla W_{i})N_{i+1} + r_i(n_i \cdot \nabla W_i)N_i) \, \end{aligned}$$

Again approximating the time differential by finite differences, we get a system of the following form for $Y_i^j$:

44 $$\begin{aligned} A_i^j Y_{i-2}^j + B_i^j Y_{i-1}^j + C_i^j Y_i^j + D_i^j Y_{i+1}^j + E_i^j Y_{i+2}^j = F_i^j. \end{aligned}$$

The coefficients of the linear equations (40), (42) and (44) are reported in the following.

#### Discretisation of the Equation for *K*

In the following, we report how the different terms appearing in Eq. (39) have been discretised

$$\begin{aligned}{}[\partial _\ell ^3 K]_{i-1}^i&\approx \left[ \frac{\partial _\ell ^2 K ({\tilde{Y}}_{m+1})-\partial _\ell ^2 K ({\tilde{Y}}_{m})}{q_m} \right] _{i-1}^i \\&\approx \Bigg [ \frac{1}{q_m} \left( \frac{\partial _\ell K (Y_{m+1})-\partial _\ell K (Y_{m})}{r_{m+1}} \right) \\&\quad - \frac{1}{q_i} \left( \frac{\partial _\ell K (Y_{m})-\partial _\ell K (Y_{m-1})}{r_{m}} \right) \Bigg ]_{i-1}^i \\&\approx \Bigg [ \frac{1}{q_m r_{m+1}} \left( \frac{K_{m+2}-K_{m+1}}{q_{m+1}} - \frac{K_{m+1}-K_{m}}{q_{m}} \right) \\&\quad -\frac{1}{q_m r_{m}} \left( \frac{K_{m+1}-K_{m}}{q_{m}} - \frac{K_m-K_{m-1}}{q_{m-1}} \right) \Bigg ]_{i-1}^i \\ [\partial _\ell K^3]_{i-1}^i&\approx \left[ \frac{K_{m+1}^3 - K_{m}^3}{q_m} \right] _{i-1}^i \\ [\alpha K]_{i-1}^i&\approx [\alpha _m {{\tilde{K}}}_m]_{i-1}^i = \left[ \alpha _m \frac{K_{m+1}+K_{m}}{2}\right] _{i-1}^i \end{aligned}$$

$$\begin{aligned}{}[\partial _\ell (\nabla W\cdot N)]_{i-1}^i&\approx \left[ \frac{1}{q_m}(\nabla {\tilde{W}}_{m+1} \cdot N_{m+1} - \nabla {\tilde{W}}_{m} \cdot N_{m})\right] _{i-1}^i \\&= \Bigg [\frac{1}{2q_m}((\nabla W_{m+1} + \nabla W_m) \cdot N_{m+1} \\&\qquad - (\nabla W_{m} + \nabla W_{m-1})\cdot N_{m})\Bigg ]_{i-1}^i \\ [\partial _\ell (KW)]_{i-1}^i&\approx \left[ \frac{{\tilde{W}}_{m+1}K_{m+1} - {\tilde{W}}_{m} K_{m}}{q_m} \right] _{i-1}^i \\&= \left[ \frac{1}{2 q_m} \left( \left( W_{m+1}+W_{m}\right) K_{m+1} - \left( W_{m}+W_{m-1} \right) K_{m} \right) \right] _{i-1}^i \end{aligned}$$

Therefore, the coefficients of (40) are given as follows:

$$\begin{aligned} a_i^j&= \dfrac{k_b}{q_{i-2}^j r_{i-1}^j q_{i-1}^j} \, , \quad e_i^j = \dfrac{k_b}{q_{i}^j r_{i+1}^j q_{i+1}^j} \\ b_i^j&= -k_b \left( \dfrac{1}{q_{i-1}^j r_{i}^j q_{i}^j} + \dfrac{1}{ (q_{i-1}^j)^2 r_{i}^j }+ \dfrac{1}{ (q_{i-1}^j)^2 r_{i-1}^j} + \dfrac{1}{q_{i-1}^j r_{i-1}^j q_{i-2}^j} \right) \\&\quad + \dfrac{\alpha _{i-1}^j}{2} - \dfrac{1}{2 q_{i-1}^j} (W_{i-1}^{j-1}+W_{i-2}^{j-1}) \\ d_i^j&= -k_b \left( \dfrac{1}{q_{i}^j r_{i+1}^j q_{i+1}^j} + \dfrac{1}{ (q_{i}^j)^2 r_{i+1}^j }+ \dfrac{1}{ (q_{i}^j)^2 r_{i}^j} + \dfrac{1}{q_{i}^j r_{i}^j q_{i-1}^j} \right) \\&\quad - \dfrac{\alpha _{i}^j}{2} - \dfrac{1}{2 q_{i}^j} (W_{i+1}^{j-1}+W_{i}^{j-1}) \\ c_i^j&= \dfrac{r_i^j}{\Delta t} + k_b \left( \dfrac{1}{ (q_{i}^j)^2 r_{i+1}^j } + \dfrac{1}{ (q_{i}^j)^2 r_{i}^j} + \dfrac{1}{ (q_{i-1}^j)^2 r_{i}^j} + \dfrac{1}{ (q_{i-1}^j)^2 r_{i-1}^j} + \dfrac{2}{q_{i}^j r_{i}^j q_{i-1}^j} \right) \\&\quad + \dfrac{\alpha _{i}^j}{2} - \dfrac{\alpha _{i-1}^j}{2}+ r_i^{j-1} k_i^{j-1} \beta _i^{j-1} + \dfrac{1}{2}\left( \dfrac{1}{q_i^j}+\dfrac{1}{q_{i-1}^j} \right) (W_i^{j-1}+W_{i-1}^{j-1}) \\ f_i^j&= \dfrac{r_i^j}{\Delta t} k_{i}^{j-1} - k_b \dfrac{ (k_{i+1}^{j-1})^3 - (k_{i}^{j-1})^3 }{2 q_{i}^{ j-1}} + k_b \dfrac{ (k_i^{j-1})^3 - (k_{i-1}^{j-1})^3 }{2 q_{i-1}^{j-1}} \\&\quad +\dfrac{\nabla W_{i+1}^{j-1} + \nabla W_{i}^{j-1}}{2q_i^{ j-1}} \cdot N_{i+1}^{j-1} + \dfrac{\nabla W_{i-1}^{j-1} + \nabla W_{i-2}^{j-1}}{2q_{i-1}^{ j-1}} \cdot N_{i-1}^{j-1} \\&\quad -\left( \dfrac{1}{2q_i^{j-1}} +\dfrac{1}{2q_{i-1}^{j-1}}\right) (\nabla W_{i}^{j-1}+ \nabla W_{i-1}^{j-1}) \cdot N_i^{j-1} \end{aligned}$$

#### Discretization of the Equation for $\nu$

In the following, we report how the different terms appearing in Eq. (41) have been discretized

$$\begin{aligned}{}[(\partial _\ell \nu )^3]_{i-1}^i&\approx \left[ (\frac{1}{q_m}(\nu _{m+1} - \nu _{m}))^3\right] _{i-1}^i\\ [\nabla W \cdot N]_{i-1}^i&\approx \left[ \nabla W_m \cdot \widetilde{N_m} \right] _{i-1}^i = \left[ \frac{1}{2} \nabla W_m \cdot \left( N_{m+1} + N_{m} \right) \right] _{i-1}^i \\ [\alpha \nu ]_{i-1}^i&\approx \left[ \alpha _{m}{\tilde{\nu }}_{m} \right] _{i-1}^i = \left[ \frac{1}{2} \alpha _{m} (\nu _{m}+ \nu _{m+1} )\right] _{i-1}^i \\ [W \partial _\ell \nu ]_{i-1}^i&\approx \left[ \frac{ W_m}{q_m}(\nu _{m+1}-\nu _m)\right] _{i-1}^i \end{aligned}$$

Then, the coefficients of (42) are

$$\begin{aligned} {\mathcal {A}}_i^j&= \dfrac{k_b}{q_{i-2}^j r_{i-1}^j q_{i-1}^j} \, , \quad {\mathcal {E}}_i^j = \dfrac{k_b}{q_{i}^j r_{i+1}^j q_{i+1}^j} \, ,\\ \quad {\mathcal {B}}_i^j&=-k_b \left( \dfrac{1}{q_{i-1}^j r_{i}^j q_{i}^j} + \dfrac{1}{ (q_{i-1}^j)^2 r_{i}^j }+ \dfrac{1}{ (q_{i-1}^j)^2 r_{i-1}^j} + \dfrac{1}{q_{i-1}^j r_{i-1}^j q_{i-2}^j} \right) \\&\quad + \dfrac{\alpha _{i-1}^j}{2} - \dfrac{W_{i-1}^{j-1}}{q_{i-1}^j}\, , \\ {\mathcal {D}}_i^j&= -k_b \left( \dfrac{1}{q_{i}^j r_{i+1}^j q_{i+1}^j} + \dfrac{1}{ (q_{i}^j)^2 r_{i+1}^j }+ \dfrac{1}{ (q_{i}^j)^2 r_{i}^j} + \dfrac{1}{q_{i}^j r_{i}^j q_{i-1}^j} \right) - \dfrac{\alpha _{i}^j}{2} - \dfrac{W_i^{j-1}}{q_{i}^j} \, ,\\ {\mathcal {C}}_i^j&= \dfrac{r_i^j}{\Delta t} + k_b \left( \dfrac{1}{ (q_{i}^j)^2 r_{i+1}^j } + \dfrac{1}{ (q_{i}^j)^2 r_{i}^j} + \dfrac{1}{ (q_{i-1}^j)^2 r_{i}^j} + \dfrac{1}{ (q_{i-1}^j)^2 r_{i-1}^j} + \dfrac{2}{q_{i}^j r_{i}^j q_{i-1}^j} \right) \\&\quad + \dfrac{\alpha _{i}^j}{2} - \dfrac{\alpha _{i-1}^j}{2} + \left( \dfrac{W_i^{j-1}}{q_{i}^j} + \dfrac{W_{i-1}^{j-1}}{q_{i-1}^j}\right) \\ {\mathcal {F}}_i^j&= \dfrac{r_i^j}{\Delta t} \nu _{i}^{j-1} - \dfrac{k_b}{2} \left( \dfrac{ \nu _{i+1}^{j-1} - \nu _{i}^{j-1}}{q_{i}^{ j-1}} \right) ^3 + \dfrac{k_b}{2} \left( \dfrac{ \nu _i^{j-1} - \nu _{i-1}^{j-1} }{q_{i-1}^{j-1}} \right) ^3 \\&\quad + \frac{1}{2}\nabla W_{i}^{j-1} \cdot (N_{i+1}^{j-1} + N_i^{j-1}) + \frac{1}{2}\nabla W_{i-1}^{j-1} \cdot (N_{i}^{j-1} + N_{i-1}^{j-1}) \end{aligned}$$

#### Discretisation of the Equation for $Y_i$

In the following, we report how the different terms appearing in Eq. (43) have been discretised

$$\begin{aligned} W_i [\partial _\ell Y]_{{\widetilde{i}}}^{\widetilde{i+1}} = W_i \left[ \frac{Y_{m}-Y_{m-1}}{r_{m}}\right] _{i}^{i+1} \end{aligned}$$

The coefficients of (44) are

$$\begin{aligned} A_i^j&= \dfrac{k_b}{r_{i-1}^j q_{i-1}^j r_{i}^j} \, , \quad E_i^j = \dfrac{k_b}{r_{i+1}^j q_{i+1}^j r_{i+2}^j} \\ B_i^j&= -k_b \left( \dfrac{1}{r_{i-1}^j q_{i-1}^j r_{i}^j} + \dfrac{1}{ (r_{i}^j)^2 q_{i-1}^j }+ \dfrac{1}{ (r_{i}^j)^2 q_{i}^j} + \dfrac{1}{r_{i}^j q_{i}^j r_{i+1}^j} \right) \\&\quad + \dfrac{3}{2}k_b \dfrac{(k_i^j)^2}{r_i^j}+\dfrac{\alpha _{i}^j}{2} - \dfrac{W_i^{j-1}}{r_i^j} \\ D_i^j&= -k_b \left( \dfrac{1}{r_{i}^j q_{i}^j r_{i+1}^j} + \dfrac{1}{ (r_{i+1}^j)^2 q_{i}^j }+ \dfrac{1}{ (r_{i+1}^j)^2 q_{i+1}^j} + \dfrac{1}{r_{i+1}^j q_{i+1}^j r_{i+2}^j} \right) \\&\quad + \dfrac{3}{2}k_b \dfrac{(k_{i+1}^j)^2}{r_{i+1}^j} - \dfrac{\alpha _{i}^j}{2} - \dfrac{W_i^{j-1}}{r_{i+1}^j} \\ C_i^j&= \dfrac{ q_i^j}{\Delta t} - \left( A_i^j + B_i^j +D_i^j + E_i^j\right) \\ F_i^j&= \dfrac{q_i^j}{\Delta t} y_{i}^{j-1} - \frac{\Delta p_n + \mu _n (A_n -A_{n}^*) }{2} \left( r_{i+1}^{j-1} N_{i+1}^{j-1} + r_i^{j-1} N_i^{j-1} \right) \\&\quad -\, \frac{1}{2}(r_{i+1}^{j-1}(N_{i+1}^{j-1} \cdot \nabla W_{i}^{j-1})N_{i+1}^{j-1} + r_i^{j-1}(N_i^{j-1} \cdot \nabla W_i^{j-1})N_i^{j-1}) \, \end{aligned}$$

We remark that the linear dependency on *Y* inside *W*(*Y*) can be possibly treated implicitly.

## Footnotes

1 Jensen’s inequality states that $\phi \left( \frac{1}{{\vert a-b \vert }}\int _a^b f(x) \; dx\right) \le \frac{1}{{\vert a-b \vert }}\int _a^b \phi \left( f(x) \right) dx$ for any convex function $\phi : {\mathbb {R}} \rightarrow {\mathbb {R}}$ and $f \in L^1(a,b)$, see e.g. Evans (2010, B, Theorem 2).

2 julialang.org.
